# Estimation of the Mechanism of Adrenal Action of Endocrine-Disrupting Compounds Using a Computational Model of Adrenal Steroidogenesis in NCI-H295R Cells

**DOI:** 10.1155/2016/4041827

**Published:** 2016-02-17

**Authors:** Ryuta Saito, Natsuko Terasaki, Makoto Yamazaki, Naoya Masutomi, Naohisa Tsutsui, Masahiro Okamoto

**Affiliations:** ^1^Graduate School of Bioresource and Bioenvironmental Sciences, Kyushu University, Higashi-ku, Fukuoka 812-8582, Japan; ^2^Biology Research Laboratories, Mitsubishi Tanabe Pharma Corporation, Toda-shi, Saitama 335-8505, Japan; ^3^DMPK Research Laboratories, Mitsubishi Tanabe Pharma Corporation, Toda-shi, Saitama 335-8505, Japan; ^4^Safety Research Laboratories, Mitsubishi Tanabe Pharma Corporation, Kisarazu-shi, Chiba 292-0818, Japan

## Abstract

Adrenal toxicity is one of the major concerns in drug development. To quantitatively understand the effect of endocrine-active compounds on adrenal steroidogenesis and to assess the human adrenal toxicity of novel pharmaceutical drugs, we developed a mathematical model of steroidogenesis in human adrenocortical carcinoma NCI-H295R cells. The model includes cellular proliferation, intracellular cholesterol translocation, diffusional transport of steroids, and metabolic pathways of adrenal steroidogenesis, which serially involve steroidogenic proteins and enzymes such as StAR, CYP11A1, CYP17A1, HSD3B2, CYP21A2, CYP11B1, CYP11B2, HSD17B3, and CYP19A1. It was reconstructed in an experimental dynamics of cholesterol and 14 steroids from an* in vitro* steroidogenesis assay using NCI-H295R cells. Results of dynamic sensitivity analysis suggested that HSD3B2 plays the most important role in the metabolic balance of adrenal steroidogenesis. Based on differential metabolic profiling of 12 steroid hormones and 11 adrenal toxic compounds, we could estimate which steroidogenic enzymes were affected in this mathematical model. In terms of adrenal steroidogenic inhibitors, the predicted action sites were approximately matched to reported target enzymes. Thus, our computer-aided system based on systems biological approach may be useful to understand the mechanism of action of endocrine-active compounds and to assess the human adrenal toxicity of novel pharmaceutical drugs.

## 1. Introduction

Because steroid hormones play an important role in a wide range of physiological processes, the potential to disturb endocrine effects is a major concern in the development of novel pharmaceutical drugs such as etomidate and aminoglutethimide [1]. The adrenal gland is the most common target for toxicity in the endocrine system* in vivo*, because steroid hormones are primarily synthesized through enzymatic reactions in the adrenal cortex [2–5]. Indeed, in these studies based on chemically induced endocrine lesions observed* in vivo*, the most frequent site of reported effects was the adrenal gland. Therefore, the prediction of human adrenal toxicity based on the mechanism of on- or off-target actions in the early stages of drug development is important.

The NCI-H295R human adrenocortical carcinoma cell line has been used to elucidate mechanisms of adrenal steroidogenic disrupting compounds [1, 6]. The H295R cell line was established by Gazder and his collaborators in 1990 [7], which expresses all key steroidogenic enzymes and steroidogenesis-related proteins [7–9]. H295R cells have the physiological characteristics of zonally undifferentiated human fetal adrenal cells and the ability to produce steroid hormones found in the adult adrenal cortex [1, 7, 9].* In vitro* bioassays using the H295R human cell line have been able to evaluate the effects of chemicals on steroid hormone production [10–15], steroidogenic enzyme activities [11, 16, 17], and the expression of steroidogenic genes [11, 18]. In transcriptome studies, the mechanisms of action of many steroidogenic disrupting compounds have been qualitatively assessed in terms of adrenal toxicity. However, gene expression does not always reflect the production of steroid hormones [19]. Furthermore, measuring a few specific steroid hormones may not be a useful approach to study the mechanisms of steroidogenic disrupting effects in complex pathways such as adrenal steroidogenesis. To systematically understand how exogenous compounds affect adrenal steroidogenesis, simultaneous determination of all detectable steroid hormones and integrative analysis of these complex data would be important. As an exploratory approach to analyze complex data, ToxClust developed by Zhang and colleagues in 2009 is able to visualize concentration-dependent response relationships in the characteristics of chemically induced toxicological effects [20]. However, this exploratory approach is unable to provide a quantitative understanding of the mechanism of action of adrenal toxicants or reveal systematic information about the effect of each enzymatic reaction, interactions, and feedback in the adrenal steroidogenesis pathway.

Systems biology based on computational models of biological processes and the comprehensive measurement of biological molecules is the most powerful approach to quantitatively understand the influence of each factor in complex biological pathways. In recent studies by our collaborators, a computational model of adrenal steroidogenesis has been developed in NCI-H295R cells, including the steroidogenic disrupting effects of metyrapone to inhibit enzymatic reactions of CYP11B1 [21, 22]. The model reproduces the dynamics of adrenal steroidogenesis in NCI-H295R cells and the influence of metyrapone. A current computational model of adrenal steroidogenesis was incorporated with a reaction of oxysterol synthesis as a bypass to consume cellular cholesterol [22]. In addition, all reactions in this model are described by a kinetic equation of the first-order reaction [22]. It is difficult to quantitatively evaluate the influence of each protein in the complicated system of adrenal steroidogenesis using the reported models, because it is simple and any biochemical and cellular biological information is not sufficient. For example, to clearly understand the cause of the change from the differentially dynamic patterns of steroid hormones, it is necessary to consider the substrate inhibition of steroidogenic enzyme because most of steroidogenic enzymes recognize multiple steroids as the enzymatic substrate. However, the substrate inhibition of steroidogenic enzyme cannot be described by the mathematical model based on kinetic equations of first-order reaction that does not consider Michaelis constant *K*_*m*_ expressing the affinity of the substrate. To quantitatively estimate the mechanism of steroidogenic disrupting compounds from comprehensive experimental data of adrenal steroidogenesis in NCI-H295R cells, the reported model should be improved according to the following two points. First, the kinetic equation of enzymatic reactions should be exchanged from the first-order equation to a steady-state kinetic equation based on the mechanism of the enzymatic reaction. Because a mathematical model organized by first-order equations operates in a simple structure-dependent manner, it does not show complex behavior based on molecular interactions, feedback, or regulation. Second, intracellular localization processes of cholesterol should be incorporated as a considerable mechanism. Because intracellular cholesterol molecules are stored as cholesterol esters or widely distributed as membrane components, only a few cholesterol molecules localized on the mitochondrial inner membrane are available for the adrenal steroidogenesis pathway [23, 24]. Moreover, cholesterol-trafficking processes from the outer to inner mitochondrial membranes, which are regulated by steroidogenic acute regulatory (StAR) protein, are one of the rate-limiting steps in adrenal steroidogenesis [24]. By overcoming these limitations in the reported steroidogenesis model, systems analysis of adrenal steroidogenesis in H295R cells may be able to quantitatively estimate the mechanism of action of steroidogenic disrupting compounds.

In the present study, to quantitatively estimate the toxicological mechanism of endocrine-active compounds in adrenal steroidogenesis and to predict human adrenal toxicity of novel pharmaceutical drugs in the drug discovery phase, we developed a novel computational model of steroidogenesis in NCI-H295R cells. It includes cholesterol transport into intracellular regions from the extracellular space, the cholesterol translocation system in intracellular regions, including oxysterol synthesis, the metabolic pathway of adrenal steroidogenesis, and transport of steroid hormones. Global sensitivity analysis of this adrenal steroidogenesis model is able to evaluate the influence of each steroidogenic enzyme and related protein for each steroid hormone observed in an* in vitro* steroidogenesis assay of NCI-H295R cells. Furthermore, the mechanisms of action of steroidogenesis disrupting compounds for steroidogenic enzymes can be estimated by the optimization method to solve the reverse problem from the concentration changes of 12 steroid hormones measured by liquid chromatography/mass spectrometry in the steroidogenesis assay of NCI-H295R cells* in vitro*. Using this developed model of adrenal steroidogenesis and the analytical approach, the* in vitro* steroidogenesis assay of NCI-H295R cells can assess the human adrenal toxicity of a novel pharmaceutical drug based on quantitative understanding of its toxicological mechanism in adrenal steroidogenesis.

## 2. Materials and Methods

### 2.1. The Experimental Part

#### 2.1.1. Cell Culture

NCI-H295R human adrenocortical carcinoma cells were purchased from the American Type Culture Collection (Cat# CRL-2128, Manassas, VA) and cultured at 37°C in a humidified atmosphere with 5% CO_2_. The cells were maintained in a 1 : 1 mixture of Dulbecco's modified Eagle's medium (DMEM, GIBCO, Life Technologies, Carlsbad, CA) and F-12 medium (MP Biomedicals Inc., Irvine, CA) supplemented with 15 mM HEPES (Dojindo Laboratories, Kumamoto, Japan), 0.00625 mg/mL insulin (Sigma-Aldrich, Inc., St. Louis, MO), 0.00625 mg/mL transferrin (Sigma-Aldrich Inc., St. Louis, MO), 30 nM sodium selenite (Wako Pure Chemical Industries Ltd., Osaka, Japan), 1.25 mg/mL bovine serum albumin (BSA, Sigma-Aldrich Inc., St. Louis, MO), 0.00535 mg/mL linoleic acid (Sigma-Aldrich Inc., St. Louis, MO), 2.5% Nu Serum (Becton, Dickinson and Company, Franklin Lakes, NJ), 100 U/mL penicillin (Meiji Seika Pharma, Tokyo, Japan), and 100 mg/L streptomycin (Meiji Seika Pharma, Tokyo, Japan).

#### 2.1.2. Adrenal Steroidogenesis in Human Adrenal Corticocarcinoma NCI-H295R Cells

NCI-H295R cells were stimulated with adrenocorticotrophic hormone (ACTH), forskolin, and angiotensin II to initiate steroidogenesis. Changes in steroid concentrations over time were measured after stimulation in both cells and culture medium to construct a simulation model.

The cells were seeded at 6 × 10^5^ cells/well in 6-well plates. After 3 days of culture, the culture medium was changed to stimulation medium consisting of DMEM/F-12 (1 : 1) medium supplemented with 0.00625 mg/mL insulin, 0.00625 mg/mL transferrin, 30 nM sodium selenite, 1.25 mg/mL BSA, 0.00535 mg/mL linoleic acid, 10% fetal bovine serum (GIBCO, Life Technologies, Carlsbad, CA), 100 U/mL penicillin, 100 mg/L streptomycin, 50 nM ACTH (Sigma-Aldrich Inc., St. Louis, MO), 20 *μ*M forskolin (Sigma-Aldrich Inc., St. Louis, MO), and 100 nM angiotensin II (Calbiochem, Merck Millipore, Darmstadt, Germany). Culture media and cells were collected at 0, 8, 24, 48, and 72 h after stimulation. The cells were collected in 100 *μ*L distilled water and sonicated to produce a cell lysate. The cultures were conducted in four wells/time point (*N* = 4).

The concentrations of 12 steroids, pregnenolone (PREG), 17*α*-hydroxypregnenolone (HPREG), dehydroepiandrosterone (DHEA), progesterone (PROG), 17*α*-hydroxyprogesterone (HPROG), androstenedione (DIONE), testosterone (TESTO), 11-deoxycorticosterone (DCORTICO), 11-deoxycortisol (DCORT), corticosterone (CORTICO), cortisol (CORT), and aldosterone (ALDO), in the medium and cell lysate were measured by LC/MS. Concentrations of estrone (E1) and 17*β*-estradiol (E2) were measured by enzyme-linked immunosorbent assays (Wako Pure Chemical Industries Ltd., Osaka, Japan). In addition, the concentration of cholesterol was measured using a commercial kit (Wako Pure Chemical Industries Ltd., Osaka, Japan) based on the cholesterol oxidase method.

#### 2.1.3. Liquid Chromatography

A LC-VP series (Shimadzu, Kyoto, Japan) consisting of an SIL-HTc autosampler, LC-10ADvp Pump, CTO-10ACvp column oven, and DGU-14AM degasser was used to set the reverse-phase liquid chromatographic conditions. The column was a Cadenza CD-C18 column (100 × 2 mm i.d., 3 *μ*m, Imtakt Corp., Kyoto, Japan) used at 45°C. The mobile phase included water/acetonitrile/formic acid 95/5/0.05 (v/v/v, Solvent A) and water/acetonitrile/formic acid 35/65/0.05 (v/v/v, Solvent B). The gradient elution programs were 0% B (0-1 min with an isocratic gradient), 0–40% B (1-2 min with a linear gradient), 40% B (2–7 min with an isocratic gradient), 40–100% B (7–12 min with a linear gradient), 100% B (12–14 min with an isocratic gradient), 100–0% B (14-15 min with a linear gradient), and 0% B (15-16 min with an isocratic gradient) at a flow rate of 0.3 mL/min. The autosampler tray was cooled to 45°C and the injection volume was 5 *μ*L. HPLC grade acetonitrile and formic acid were purchased from WAKO.

#### 2.1.4. Mass Spectrometry

A triple quadrupole mass spectrometer API4000 (Applied Biosystems/MDS Sciex, Concord, Canada) coupled with an electrospray ionization source was operated in the positive ion mode. The optimized ion source conditions were as follows: collision gas, 6 psi; curtain gas, 40 psi; ion source gas 1, 50 psi; ion source gas 2, 80 psi; ion source voltage, 5500 V; ion source temperature, 600°C. Nitrogen was used as the collision gas in the multiple reaction monitoring (MRM) mode. The conditions of declustering potential, collision energy, and collision cell exit potential were optimized by every steroid. The transitions in MRM were as follows: PREG *m*/*z* 317 → 299, HPREG* m/z* 315 → 297, DHEA* m/z* 289 → 271, PROG* m/z* 315 → 109, HPROG* m/z* 331 → 109, DIONE* m/z* 287 → 97, DCORT* m/z* 331 → 123, DCORTICO* m/z* 347 → 161, CORTICO* m/z* 347 → 100, CORT* m/z* 363 → 309, ALDO* m/z* 361 → 343, and TESTO* m/z* 289 → 109. Mass spectroscopic data were acquired and quantified using the Analyst 1.4.2 software package (Applied Biosystems/MDS Sciex, Concord, Canada).

#### 2.1.5. Estimation of the Cell Volume

Cell volume was estimated from the number of cells in the well and the average diameter of the cells. Cells were detached from the well using 0.025% trypsin (MP Biomedicals, Inc., Irvine, CA) in a 0.02% EDTA solution (Dojindo Laboratories, Kumamoto, Japan) at the start of preculture, start of stimulation, and at 24, 48, and 72 h after stimulation. The numbers and diameters of the cells were measured by a cell counter Vi-cell XR 2.01 (Beckman Coulter, Krefeld, Germany) after trypan blue staining. Parameters of the cell volume and number of cells were estimated to fit experimental time-course data using exponential curves.

#### 2.1.6. Test Compounds in Validation Study

NCI-H295R cells were exposed to seven well-characterized inhibitors of steroidogenesis, and then the concentrations of the steroids in the culture medium were measured to estimate the enzyme inhibition to evaluate the performance of the simulation model. The adrenal steroidogenic inhibitors included aminoglutethimide (AGT, Bachem AG, Bubendorf, Switzerland), o,p′-DDD (DDD, Sigma-Aldrich Inc., St. Louis, MO), spironolactone (SP, Sigma-Aldrich Inc., St. Louis, MO), metyrapone (MP, Sigma-Aldrich Inc., St. Louis, MO), ketoconazole (KC, Wako Pure Chemical Industries, Ltd., Osaka, Japan), miconazole (MC, Wako Pure Chemical Industries, Ltd., Osaka, Japan), and daidzein (DZ, Sigma-Aldrich Inc., St. Louis, MO). The cells were also exposed to four adrenal toxicants whose adrenal toxicity is not mediated through steroidogenesis inhibition. The toxicants were acrylonitrile (AN, Wako Pure Chemical Industries, Ltd., Osaka, Japan), salinomycin (SM, Sigma-Aldrich, Inc., St. Louis, MO), thioguanine (TG, Tokyo Chemical Industry Co., Ltd., Tokyo, Japan), and fumaronitrile (FN, Wako Pure Chemical Industries, Ltd., Osaka, Japan). All chemicals were dissolved in DMSO (Wako Pure Chemical Industries, Ltd., Osaka, Japan) and added to the culture medium at 1 : 1000 dilutions.

#### 2.1.7. Validation Study Using Adrenal Toxicants

NCI-H295R cells were cultured for 3 days in 6-well plates and then stimulated with the above-mentioned compounds. Upon the start of stimulation, various concentrations of test chemicals were added to the cultures. After a further 3 days of culture with the chemicals, the concentrations of 12 steroids (PREG, HPREG, DHEA, PROG, HPROG, DIONE, DCORTICO, DCORT, CORTICO, CORT, ALDO, and TESTO) in the culture medium were measured by LC/MS/MS. The test concentrations of the chemicals were determined by dose-finding cytotoxicity assays. The cytotoxicity assay was conducted in 96-well plates using ATP content in cells as an endpoint (CellTiter-Glo*™* Luminescent Cell Viability Assay, Promega). Concentrations that caused more than 20% cytotoxicity were not used in the steroidogenesis assay. The test concentrations of adrenal steroidogenesis inhibitors and other compounds are shown in Table 1.

#### 2.1.8. Statistical Analysis

Comparisons were performed by the two-sample Welch's *t*-test with Bonferroni multiple testing correction for each steroid hormone species. Statistically significant steroid hormones were considered at adjusted *p* values of less than 0.01. Differential metabolic steroid profiles were classified by hierarchical cluster analysis. Pairwise distances between all compounds and all steroids were calculated by standardized Euclidean metric. This distance matrix was analyzed with Ward's method for hierarchical clustering. Statistical analysis was performed using MATLAB software (MathWorks, Inc., Natick, MA).

### 2.2. The Computational Part

#### 2.2.1. Mathematical Modeling of Adrenal Steroidogenesis in NCI-H295R Cells

**Figure 1:**
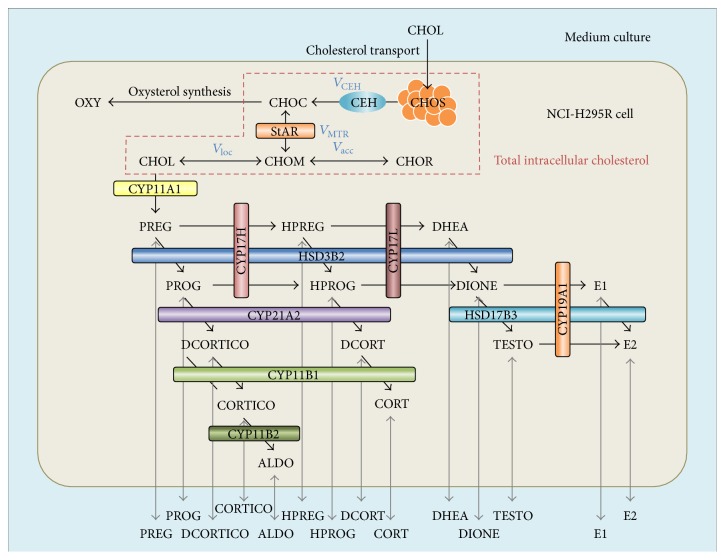
Schematic diagram of the mathematical model of adrenal steroidogenesis in NCI-H295R cells. Overview of the mathematical model of adrenal steroidogenesis in NCI-H295R cells, including cholesterol transport and intracellular localization, oxysterol synthesis, the C_21_-steroid hormone biosynthesis pathway, passive diffusional transport of steroid hormones, and cell proliferation. CHOL: total cholesterol in medium culture, CHOS: stored cholesterol esters in the endoplasmic reticulum, CHOC: intracellular free cholesterol, CHOM: mitochondrial free cholesterol, CHON: mitochondrial free cholesterol close to CYP11A1 enzymes, CHOR: mitochondrial free cholesterol remote from CYP11A1 enzymes, PREG: pregnenolone, HPREG: 17*α*-hydroxypregnenolone, DHEA: dehydroepiandrosterone, PROG: progesterone, HPROG: 17*α*-hydroxyprogesterone, DIONE: androstenedione, DCORTICO: 11-deoxycorticosterone, DCORT: 11-deoxycortisol, CORTICO: corticosterone, CORT: cortisol, ALDO: aldosterone, TESTO: testosterone, E1: estrone, E2: 17*β*-estradiol, OXY: oxysterol, CEH: cholesterol ester hydrolase, StAR: steroidogenic acute regulatory protein, CYP11A1: P450 side chain cleavage enzyme, CYP17H: 17*α*-hydroxylase of CYP17, CYP17L: C_17–20_ lyase of CYP17, HSD3B2: 3*β*-hydroxysteroid dehydrogenase, CYP21A2: 21-hydroxylase, CYP11B1: 11*β*-hydroxylase, CYP11B2: 18-hydroxylase, HSD17B3: 17*β*-hydroxysteroid dehydrogenase, and CYP19A1: aromatase.

Steroid hormones secreted from human adrenal corticocarcinoma NCI-H295R cells are synthesized from cholesterol through the C_21_-steroid hormone biosynthesis pathway. A mathematical model of adrenal steroidogenesis in NCI-H295R cells was constructed with cholesterol transport and the intracellular localization pathway, the oxysterol synthesis pathway as a bypass of steroidogenesis, the C_21_-steroid hormone biosynthesis pathway as the main steroidogenesis pathway, passive transport of steroid hormones, and cell proliferation (Figure 1). In this model, two compartments, the intracellular space and culture medium, were incorporated as the available region. Equations and parameters of the cell proliferation and diffusional transport of steroid hormones have been proposed by previous studies [21, 22]. Cholesterol transport and the intracellular localization pathway including the oxysterol bypass were integrated using a part of the ACTH-stimulated cortisol secretion model described by Dempsher and colleagues [47]. The C_21_-steroid hormone biosynthesis pathway includes 14 steroid hormones, PREG, HPREG, DHEA, PROG, HPROG, DIONE, TESTO, DCORTICO, DCORT, CORTICO, CORT, ALDO, E1, and E2, and 17 enzymatic reactions catalyzed by nine steroidogenic enzymes, cholesterol side chain cleavage enzyme (CYP11A1), 17*α*-hydroxylase (CYP17H), C_17,20_-lyase (CYP17L), 3*β*-hydroxysteroid dehydrogenase (HSD3B2), 21-hydroxylase (CYP21A2), 11*β*-hydroxylase (CYP11B1), 18-hydroxylase (CYP11B2), 17*β*-hydroxysteroid dehydrogenase (HSD17B3), and aromatase (CYP19A1). In this mathematical model of adrenal steroidogenesis in NCI-H295R cells, the flux velocities of molecular transportation and enzymatic reaction rates of steroidogenic enzymes were defined based on the first-order reaction and rapid-equilibrium enzyme kinetics, respectively. All equations in the mathematical model of adrenal steroidogenesis of NCI-H295R cells were described in a supplementary document (see Supplementary Material available online at http://dx.doi.org/10.1155/2016/4041827). The rate constants and the maximum activities were estimated by fitting to experimental time-course data of the concentrations of cholesterol and all steroids. Initial values of cholesterol and the 14 steroid concentrations were used in each experimentally measured value, and every steroid concentration was assumed to rapidly reach the equilibrium state between the culture medium and intracellular space. All fixed values of static parameters and initial values of variable parameters in this model were described in Tables S1 and S2 in a supplementary document, respectively.

#### 2.2.2. Modeling and Simulation Environment

This computational model of adrenal steroidogenesis in NCI-H295R cells was developed on the* simBio* platform which is a general environment of biological dynamic simulation and computational model development [48]. ODEs were solved by the fourth-order Runge-Kutta method with a variable time step. The time step (*dt*) was adjusted to refer to the maximum absolute value of flux velocities or enzymatic reaction rates at each time point, and the range of the time step was from 1 × 10^−5^ to 10^−2^. To confirm whether the range of the time step was suitable, the numerical error ratio was calculated by certain fixed time steps in the range of the time step, which was under 1 × 10^−8^ in every time step. The duration time of computational simulation of adrenal steroidogenesis in NCI-H295R cells was set at 72 h.

#### 2.2.3. Parameter Optimization

To reconstruct experimental time-course patterns of the concentrations of cholesterol and the 14 steroids in the culture medium and intracellular space, we optimized every rate constant and maximum velocity of the steroidogenic enzymes. This parameter optimization problem was solved by the Levenberg-Marquardt method which is one of the nonlinear least squares methods [49–51]. The objective function of optimization was used as the following normalized least squares distance (NLSD):

(1) $$\begin{array}{c} \text{NLSD}=\underset{h}{\sum }\;\underset{i}{\sum }\;\underset{j}{\sum }\frac{{\left(X_{h,i,j}^{exp}-X_{h,i,j}^{sim}\right)}^{2}}{{X_{h,i}^{max}}^{2}}, \end{array}$$

where *h* is the compartment (culture medium or intracellular space), *i* is the molecular species (cholesterol and the 14 steroids), *j* is the time point (0, 8, 24, 48, and 72 h), *X*_*h*,*i*,*j*_^exp^ is the experimentally measured concentration of molecule *i* in compartment *h* at time point *j*, *X*_*h*,*i*,*j*_^sim^ is the simulated concentration of molecule *i* in compartment *h* at time point *j*, and *X*_*h*,*i*_^max^ is the maximum concentration of molecule *i* in compartment *h* over all time points. Data points under the lower quantitation limit were excluded from the evaluation by the objective function.

Effects of every static model parameter for parameter optimization were calculated from differences of fitting the objective function using sensitivity analysis.

#### 2.2.4. Quantitative Estimation of the Mechanism of Action of Adrenal Toxicants

Metabolic steroid profiling and differential patterns of the adrenal steroid hormones by chemical perturbation were reconstructed to optimize the relative activities of the steroidogenic enzymes. The input data for the quantitative mechanistic analysis of adrenal toxic compounds was a fold change (ratio) of the measured 12 steroid concentrations induced by drug exposure for 72 h. The two-step optimization method of the real-coded genetic algorithm (RCGA) was adopted as a global optimization method in the quantitative mechanistic analysis of adrenal toxic compounds. The operations of the crossover and generation alteration model in RCGA were used for the real-coded ensemble crossover (REX) and just generation gap (JGG) [52–55]. As the initial parameters of RCGA, maximum generation, population size, selection size of parent individuals, population size of child individuals, and termination criteria were 1000, 100, 6, 25, and under 0.1 of NLSD, respectively. The search space for the relative activities of the steroidogenic enzymes was from 1/100 to 100. To evaluate the fitness of each individual, the sum of squared residuals for fold changes of measured 12 steroid concentrations was used as the objective function. Nonlinear least squares optimization by the Levenberg-Marquardt method was used as a local search [49–51]. As the estimated mechanisms of actions of the adrenal toxic compounds, the relative activities of eight steroidogenic enzymes (CYP11A1, CYP17H, CYP17L, HSD3B2, CYP21A2, CYP11B1, CYP11B2, and HSD17B3) were optimized by the above-mentioned 2-step optimization method. Every optimization calculation was duplicated to check the numerical stability of the optimal parameters.

#### 2.2.5. Global Dynamic Sensitivity Analysis

The property of every kinetic parameter in this computational model of steroidogenesis in NCI-H295R cells was evaluated by dynamic sensitivity analysis. The sensitivity (*S*_*x*,*y*_) of kinetic parameter *x* for variable parameter *y* was defined by the following equation:

(2) $$\begin{array}{c} S_{x,y\left(t\right)}=\frac{{\Delta y}_{\left(t\right)}/y_{\left(t\right)}}{\Delta x/x}, \end{array}$$

where variable parameter *y* was the concentration of a steroid hormone in the cytosolic space of NCI-H295R cells. The perturbation for kinetic parameters was +10% (Δ*x*/*x* = 0.1).

## 3. Results

### 3.1. Experimental Data on Adrenal Steroidogenesis

#### 3.1.1. Adrenal Steroidogenesis of NCI-H295R Cells and the Mass Balance

**Figure 2:**
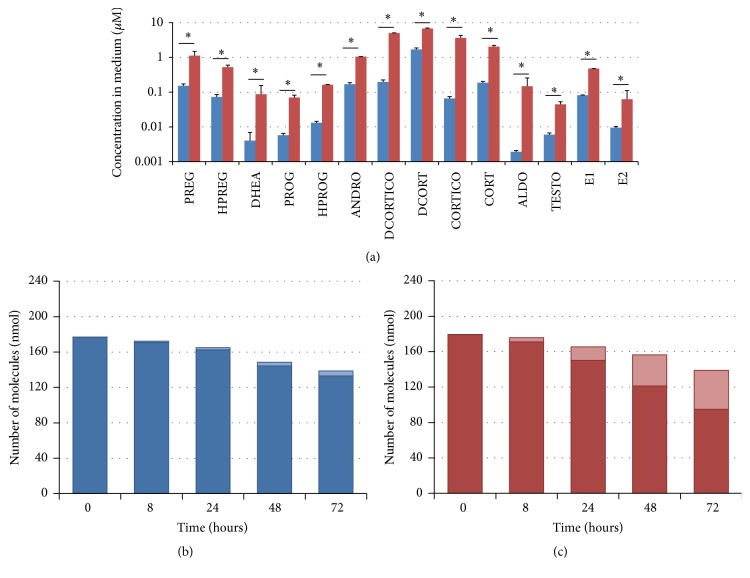
Experimental data of metabolic profiling of adrenal steroid hormones and the mass balance. Concentrations of steroid hormones secreted from NCI-H295R cells in the culture medium at 72 h after stimulation were compared with the untreated condition and the stimulated condition by 50 nM ACTH, 20 *μ*M forskolin, and 100 nM angiotensin II (a). Net molecular amounts including cholesterol and steroid hormones in the culture medium and intracellular space are plotted at five time points (0, 8, 24, 48, and 72 h after stimulation) under the untreated condition (b) and the stimulated condition (c). In the bar graphs, dark and light bars indicate the amount of cholesterol and adrenal steroids, respectively. All data are shown as the mean ± SD (*N* = 4). ^*∗*^ *p* values corrected by the familywise error rate <0.01. PREG: pregnenolone, HPREG: 17*α*-hydroxypregnenolone, DHEA: dehydroepiandrosterone, PROG: progesterone, HPROG: 17*α*-hydroxyprogesterone, DIONE: androstenedione, DCORTICO: 11-deoxycorticosterone, DCORT: 11-deoxycortisol, CORTICO: corticosterone, CORT: cortisol, ALDO: aldosterone, and TESTO: testosterone.

All steroid hormones in the culture medium were significantly increased after 72 h of stimulation with 50 nM ACTH, 20 *μ*M forskolin, and 100 nM angiotensin II (Figure 2(a)). Mass balances in steroidogenesis of NCI-H295R cells under nontreatment and control (stimulated) conditions are shown in Figures 2(b) and 2(c), respectively. Under stimulation, the dynamics of net mass in these experiments were unchanged, and accumulated cholesterol was converted to adrenal steroids.

#### 3.1.2. Cytotoxicity of Adrenal Toxicants

Viabilities of cells treated with each compound were expressed as a relative value to the ATP level of the control. Effects of AN, SM TG, FN, AGT, DDD, SP, MP, KC, MC, and DZ on cell viability were determined to be valid under 80% of the relative ATP level at 7 days after treatment. AN, SM, TG, and FN showed cytotoxicity at over 100, 1, 10, and 10 *μ*M, respectively. AGT, MP, and DZ did not affect cell viability at up to 100 *μ*M. DDD, SP, KC, and MC induced less than 80% of cell viability at over 100, 50, 100, and 25 *μ*M, respectively.

#### 3.1.3. Differentially Steroid Profiling of Adrenal Toxicants

**Figure 3:**
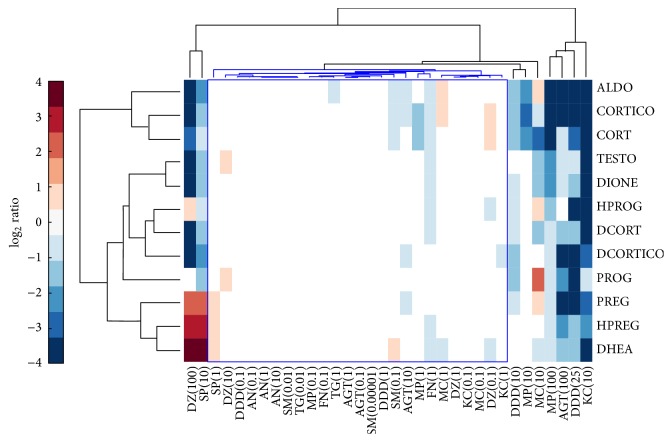
Hierarchical cluster analysis of differential metabolic profiles of 12 steroid hormones by exposure to adrenal toxicity compounds. Adrenal toxicants were classified by using the differential metabolic profiling of 12 steroid hormones. Concentrations of 12 adrenal steroids secreted from NCI-H295R cells were drastically changed by the exposure of adrenal steroidogenesis inhibitors. The 12 adrenal steroid hormones were quantitatively measured by LC-MS/MS simultaneously. Four adrenal vasculotoxic compounds: acrylonitrile: AN, fumaronitrile: FN, salinomycin: SM, and thioguanine: TG, were used as negative control compounds for adrenal steroidogenesis inhibitors. Seven adrenal steroidogenesis inhibitors: aminoglutethimide: AGT, o,p′-DDD: DDD, spironolactone: SP, metyrapone: MP, ketoconazole: KC, miconazole: MC, and daidzein: DZ, showed a characteristic steroid profile each and were classified as each independent singleton at the maximum exposure condition. Exposure concentrations of adrenal toxic compounds were described in brackets of the sample name and the units were prepared as *μ*M. A blue cluster was classified as a group of nonchange samples including negative control compounds and low exposure conditions of adrenal steroidogenesis inhibitors. PREG: pregnenolone, HPREG: 17*α*-hydroxypregnenolone, DHEA: dehydroepiandrosterone, PROG: progesterone, HPROG: 17*α*-hydroxyprogesterone, DIONE: androstenedione, DCORTICO: 11-deoxycorticosterone, DCORT: 11-deoxycortisol, CORTICO: corticosterone, CORT: cortisol, ALDO: aldosterone, and TESTO: testosterone.

After NCI-H295R cells were exposed to each test compound during three days, the concentrations of 12 steroid hormones in the culture medium were simultaneously measured by LC/MS/MS. All effects of the compounds on adrenal steroidogenesis were evaluated at the concentration without any overt cytotoxicity. The differential metabolic steroid profiles of 11 adrenal toxic compounds were classified and visualized by using hierarchical clustering analysis (Figure 3).

Four adrenal toxicants without steroidogenic inhibition, AN, SM, TG, and FN, did not change the medium concentrations of all steroid hormones by more than 2-fold. Above-mentioned 4 compounds at every condition and 7 adrenal steroidogenic inhibitors at the low exposure concentration were gathered into a big cluster as nonchange group. The 7 steroidogenic inhibitors at the maximum exposure concentration showed the characteristic steroid profiles each, but 100 *μ*M DZ and 10 *μ*M SP were classified as a cluster. AGT drastically decreased the medium concentrations of PREG, HPREG, DHEA, PROG, DCORTICO, CORTICO, and ALDO at 100 *μ*M. DDD dose-dependently decreased the medium concentrations of PROG, DCORTICO, CORTICO, CORT, and ALDO at >10 *μ*M and decreased PREG, HPREG, DHEA, PROG, HPROG, DIONE, and DCORT at the maximum exposure concentration of 25 *μ*M. SP increased PREG, HPREG, and DHEA and decreased PROG, DIONE, DCORTICO, DCORT, CORTICO, ALDO, and TESTO at 10 *μ*M. MP dose-dependently decreased CORTICO, CORT, and ALDO and decreased DHEA, HPROG, DIONE, and TESTO at the maximum exposure concentration of 100 *μ*M. KC drastically decreased the medium concentrations of PREG, HPREG, DHEA, HPROG, DIONE, DCORTICO, DCORT, CORTICO, CORTO, ALDO, and TESTO at 10 *μ*M. MC increased the medium concentrations of PROG and decreased DIONE, DCORT, CORT, and TESTO at 10 *μ*M. DZ increased PREG, HPREG, and DHEA and decreased DIONE, DCORTICO, DCORT, CORTICO, CORT, ALDO, and TESTO at 100 *μ*M.

### 3.2. The Mathematical Modeling

#### 3.2.1. Optimization of the Mathematical Model of Adrenal Steroidogenesis in NCI-H295R Cells

**Figure 4:**
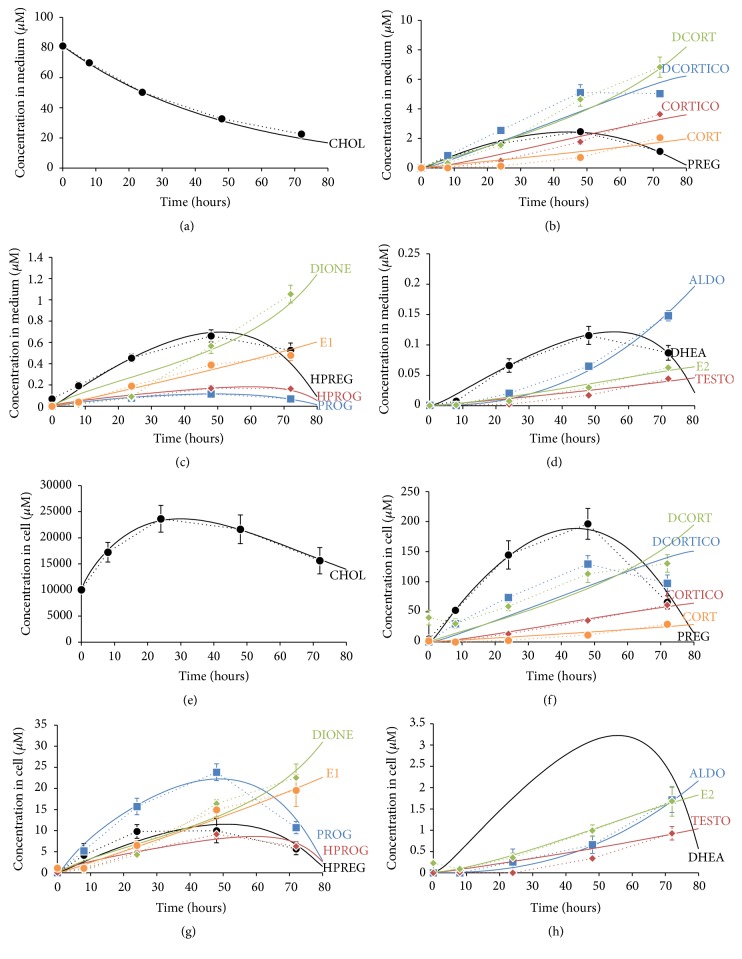
Comparison of time-course profiles of cholesterol and adrenal steroids produced by NCI-H295R cells between experimentally measured and simulated data. To intuitively confirm reconstruction of the measured experimental data in the developed simulation model of NCI-H295R cells, dynamics of cholesterol and adrenal steroids produced by NCI-H295R cells were plotted to overlay experimental data with the simulated results. Graphs show the dynamics of medium concentrations of cholesterol (a) and adrenal steroids ((b)–(d)) and intracellular concentrations of cholesterol (e) and adrenal steroids ((f)–(h)). Steroid hormones were categorized into three groups by concentration levels. Major steroids were PREG: pregnenolone, DCORTICO: 11-deoxycorticosterone, DCORT: 11-deoxycortisol, CORTICO: corticosterone, and CORT: cortisol ((b) and (f)). Moderate steroids were HPREG: 17*α*-hydroxypregnenolone, PROG: progesterone, HPROG: 17*α*-hydroxyprogesterone, DIONE: androstenedione, and E1: estrone ((c) and (g)). Minor steroids were DHEA: dehydroepiandrosterone, ALDO: aldosterone, TESTO: testosterone, and E2: 17*β*-estradiol ((d) and (h)). Experimental data are shown as symbols with dotted lines. All data represent the mean ± SD (*N* = 4). Simulation data are shown as solid lines.

The mathematical model of adrenal steroidogenesis in NCI-H295R cells was optimized for several kinetic parameters of cholesterol transport, intracellular localization, the oxysterol pathway, and maximum velocity of steroidogenic enzymes to fit the experimental time-course data. All optimized kinetic parameters are shown in Table S1 in a supplementary document. The optimized mathematical model was reconstructed with the experimental dynamic patterns of cholesterol and the 14 steroid hormones in the intracellular space and culture medium. The fitness was 0.621761 of NLSD values as the fitting objective function. The simulation results and experimental data are shown in Figure 4.

Optimized kinetic parameters were calculated sensitivities for the NLSD value as the fitting score and are shown in Table S1 in a supplementary document. The highly sensitive parameters for fitting the NLSD score were the extracted nine kinetic parameters, *k*_Cholesterol  Transport_, *k*_*f*_^acc^, *k*_*f*_^loc^, *k*_*b*_^loc^, *V*_max_^CYP11A1^, *K*_*m*_^CYP11A1^, *V*_maxA_^CYP17H^, *V*_maxA_^HSD3B2^, and *V*_maxA_^CYP21A2^, which had higher than 3.0 fitting sensitivity.

### 3.3. The Validation Using the Adrenal Toxicants

#### 3.3.1. Mechanistic Analysis of Adrenal Toxicants

**Figure 5:**
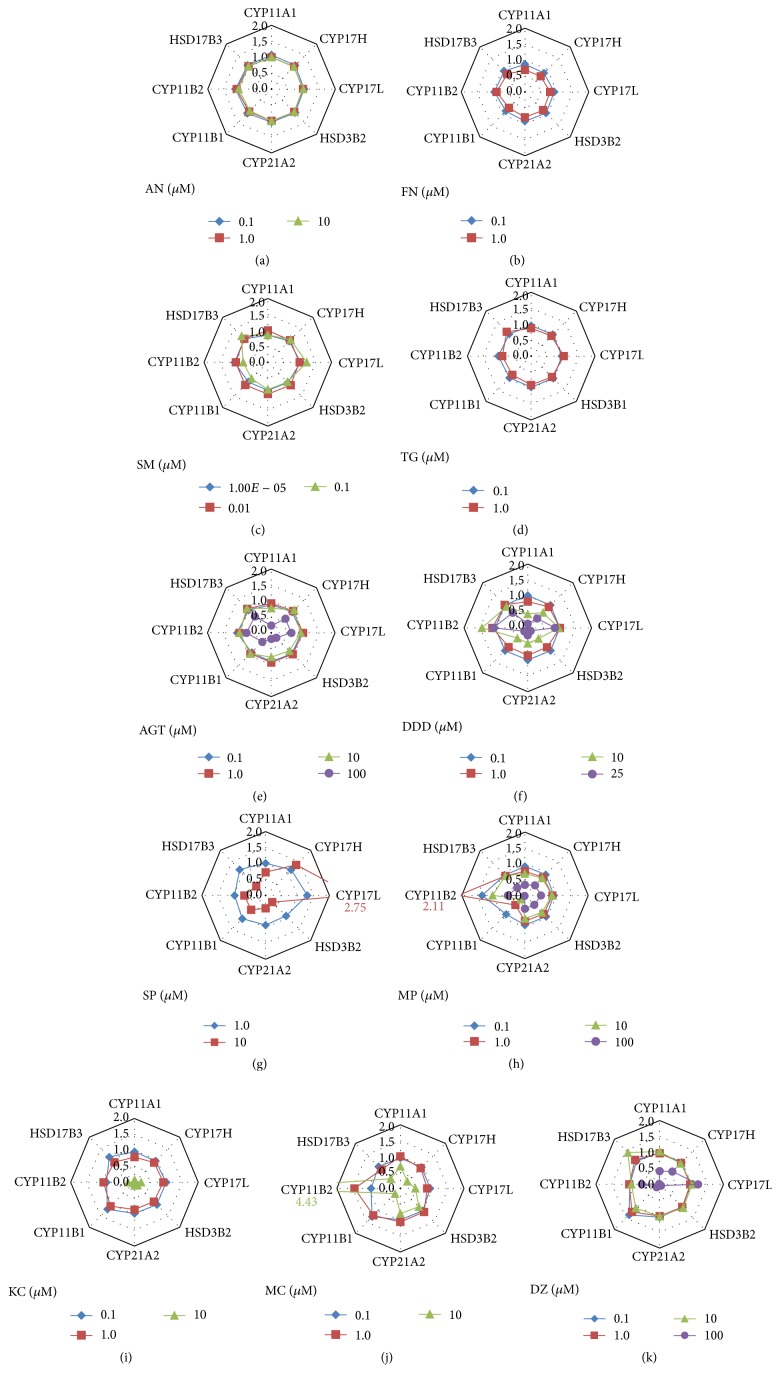
Estimated mechanism of action of adrenal toxicants by using the mathematical model of adrenal steroidogenesis in NCI-H295R cells. Mechanisms of action of adrenal toxicity compounds were quantitatively estimated from experimental results of differential steroid profiling using the mathematical model of adrenal steroidogenesis in NCI-H295R cells. The drug action was defined as a scaling factor of enzymatic activity in the simulation model. These scaling factors were optimized to fit experimental data by a hybrid optimization method of the RCGA and nonlinear least squares. Estimated drug actions by the exposure of vasculotoxic agents acrylonitrile: AN (a), fumaronitrile: FN (b), salinomycin: SM (c), and thioguanine: TG (d) and the steroidogenic inhibitors aminoglutethimide: AGT (e), o,p′-DDD: DDD (f), spironolactone: SP (g), metyrapone: MP (h), ketoconazole: KC (i), miconazole: MC (j), and daidzein: DZ (k) are shown as a spider radar chart. CYP11A1: P450 side chain cleavage enzyme, CYP17H: 17*α*-hydroxylase of CYP17, CYP17L: C_17–20_ lyase of CYP17, HSD3B2: 3*β*-hydroxysteroid dehydrogenase, CYP21A2: 21-hydroxylase, CYP11B1: 11*β*-hydroxylase, CYP11B2: 18-hydroxylase, and HSD17B3: 17*β*-hydroxysteroid dehydrogenase.

Effects of adrenal toxic compounds on steroidogenic enzymes were quantitatively predicted from the change in the ratio of the measured medium concentrations of the 12 steroid hormones at 72 h after drug exposure using the mathematical model of adrenal steroidogenesis in NCI-H295R cells. The reproducibility of the estimated results was confirmed by performing the test twice. The estimated effects of 11 adrenal toxic compounds on eight steroidogenic enzymes are shown in Figure 5. The adrenal toxic compounds without steroidogenic inhibition, such as vasculotoxic agents (AN, SM, TG, and FN), were not estimated for the target steroidogenic enzymes under noncytotoxic conditions. Every fitness values were under 0.05 of NLSD values used as the fitting objective function (Figures 5(a)–5(d)). Other steroidogenic inhibitors (AGT, DDD, SP, MP, KC, MC, and DZ) are described in detail below.

#### 3.3.2. AGT

The mechanism of action of AGT in adrenal steroidogenesis was estimated by inhibition of CYP11A1, HSD3B2, CYP21A2, and CYP11B1 at 100 *μ*M (estimated inhibitions were 77.0%, 78.0%, 81.1%, and 59.8%, resp.) (Figure 5(e)). AGT has been reported to inhibit CYP11A1, CYP21A2, CYP11B1, and CYP11B2 [6, 27–30]. Our results were mostly consistent with the previous reports. In particular, CYP11A1 appeared to be inhibited strongly by AGT. In our study, HSD3B2 inhibition of AGT was shown by mechanistic analysis based on systems biology approaches as a novel mechanism of action of AGT. Inhibition of AGT by CYP11B2 was not estimated in our study. However, the concentration of ALDO in the culture medium decreased to 3.8% of the normal stimulated condition. Inhibition of AGT by CYP11B2 has been shown using sheep adrenal homogenates as well as a human adrenal homogenate from a patient with Cushing's syndrome [30]. The activity of 18-hydroxylase induced by CYP11B2 was determined as the conversion of corticosterone to 18-hydroxycorticosterone in the previous study. The cause of the discrepancy regarding the effect of AGT on CYP11B2 was suggested to be substrate inhibition, because the intracellular concentration of CORTICO was increased by over 10 times of that in the culture medium to reach 50 *μ*M. Another possibility was poor quantitative reliability of the experimental data, because the ALDO concentration was under the lower limit of quantification at 100 *μ*M AGT. Hecker and colleagues reported that 3 *μ*M AGT decreases PREG and PROG concentrations and increases the TESTO concentration [10]. However, AGT did not increase the TESTO concentration in our study. One possibility is that the concentration of TESTO was already enhanced by about 3.3-fold through stimulation with ACTH, forskolin, and angiotensin II.

#### 3.3.3. DDD

The mechanism of action of DDD in adrenal steroidogenesis was estimated by dose-dependent inhibition of CYP11A1, HSD3B2, CYP21A2, and CYP11B1 (estimated inhibitions at 25 *μ*M were 87.0%, 86.9%, 76.9%, and 84.9%, resp.) (Figure 5(f)). DDD has been reported to inhibit CYP11A1, HSD3B2, CYP21A2, CYP11B1, and CYP11B2 [29, 31, 32]. Inhibition of DDD by CYP11B2 was not estimated in our study. However, the concentration of ALDO in the culture medium decreased to 3% of that in the normal stimulated condition. Inhibition of DDD by CYP11B2 has been shown using mitochondrial and microsomal fractions prepared by standard centrifugation procedures from a bovine adrenal cortex homogenate [32]. The cause of the discrepancy regarding the inhibition of DDD by CYP11B2 could not be explained by same effect in the case of AGT.

#### 3.3.4. SP

The mechanism of action of SP in adrenal steroidogenesis was estimated by inhibition of HSD3B2, CYP21A2, and HSD17B3 (estimated inhibitions at 10 *μ*M were 70.2%, 59.5%, and 59.3%, resp.) (Figure 5(g)). SP has been reported to inhibit CYP17H, CYP17L, CYP11B1, and CYP11B2 [6, 33–35]. The inhibitory effect of SP on the HSD3B2 enzyme was a novel mechanism of action. The main action of SP is as a mineralocorticoid receptor (MR) antagonist. SP has also been reported to exert some off-target effects by binding to androgen, glucocorticoid, and progesterone receptors [56–58]. SP has been shown to inhibit the production of ALDO and CORT from PREG induced by angiotensin II in H295R cells, but the specific MR antagonist eplerenone did not show the inhibitory effects [59]. Therefore, HSD3B2 inhibition by SP is not mediated via MR, and the action might be direct inhibition of HSD3B2 enzymes or a part of known off-target effects mediated through other nuclear hormone receptors. Regarding CYP17H and CYP17L, our results were consistent with previous reports [33, 34]. 7*α*-thiospironolactone, which is synthesized by deacetylation of SP, inhibits CYP17H and CYP17L [34]. The fact that there were no inhibitions of CYP17H or CYP17L in our study suggests that SP might not be deacetylated to 7*α*-thiospironolactone in NCI-H295R cells. Regarding CYP11B1 and CYP11B2, our results were unclear compared with a previous study. It has been shown that 30 *μ*M SP inhibits CYP11B1 and CYP11B2 in human and bovine adrenal mitochondria [35]. The cause of CYP11B1 and CYP11B2 inhibition by SP could not be determined in our study, which might be due to the lower maximum exposure concentration of SP than that in the previous report. We could not examine SP concentrations over 10 *μ*M because these concentrations were cytotoxic in NCI-H295R cells.

#### 3.3.5. MP

The mechanism of action of MP in adrenal steroidogenesis was estimated by dose-dependent inhibition of CYP11B1 (estimated inhibitions at 1, 10, and 100 *μ*M were 57.1%, 82.7%, and 98.2%, resp.) (Figure 5(h)). MP has been reported to inhibit CYP11B1 as its major effect and CYP11A1 and CYP11B2 as a weak effect [6, 29, 36–39]. The results were able to show that MP is a selective inhibitor of CYP11B1 in the previous report. However, the estimated effect of MP at a high concentration, 100 *μ*M as the maximum exposure concentration, was unclear. According to the previous report, selectivity of MP for CYP11B1/CYP11B2 is about five times [39]. In addition, 20 *μ*M MP has a slight inhibition effect on CYP11A1 in H295R cells [29].

#### 3.3.6. KC

The mechanism of action of KC in adrenal steroidogenesis was estimated by inhibition of CYP11A1, CYP17H, CYP17L, HSD3B2, CYP21A2, CYP11B1, and CYP11B2 (estimated inhibitions at 10 *μ*M were 92.6%, 94.3%, 51.8%, 83.0%, 88.2%, 97.4%, and 79.8%, resp.) (Figure 5(i)). KC has been reported to inhibit CYP11A1, CYP17H, CYP17L, HSD3B2, CYP21A2, and CYP11B1 [6, 29, 40–43]. Our results were almost consistent with the previous reports. KC inhibits CYP11A1, CYP17H, CYP21A2, and CYP11B1 in NCI-H295R cells at 10 *μ*M [29] and CYP17H, CYP17L, CYP21A2, and CYP11B1 in human adrenal mitochondria and Leydig cell microsomes at 2–5 *μ*M [60, 61]. However, KC has shown only weak inhibition of HSD3B2 and HSD17B3 in Leydig cells at the millimolar level [60, 61]. Regarding CYP11B2 and HSD17B3, we considered that these estimated inhibitions of KC did have sufficient reliability in terms of quantitative prediction precision, because ALDO and TESTO concentrations were less than the lower limit of quantification at 10 *μ*M KC.

#### 3.3.7. MC

The mechanism of action of MC in adrenal steroidogenesis was estimated by inhibition of CYP17H, CYP17L, CYP11B1, and HSD17B3 (estimated inhibitions at 10 *μ*M were 69.1%, 53.0%, 76.4%, and 57.1%, resp.) (Figure 5(j)). MC has been reported to inhibit not only CYP17H and CYP17L but also CYP11A1, CYP21A2, and CYP11B1 [41, 44, 45]. The results in the previous reports were able to estimate that MC is a CYP17 inhibitor. However, CYP11A1 inhibition by MC, probably instead of a reduction in StAR expression, was not clearly detected in our study using NCI-H295R cells, because there were no decreases in the concentrations of PREG and PROG in the culture medium. Indirect inhibition of CYP11A1 via the peripheral-type benzodiazepine receptor has been reported in mouse adrenocortical Y-1 cells treated with MC in the absence of stimuli by measuring PREG production [44]. On the other hand, reductions of StAR protein expression and/or transport activity without affecting total steroid synthesis or CYP11A1 and HSD3B2 enzyme expression or activities have been reported in (BU)_2_cAMP-stimulated MA-10 Leydig tumor cells treated with MC by measuring PROG production [45]. Therefore, the effect of MC on the initial reaction in adrenal steroidogenesis from cholesterol should be different according to the cell type and stimulation condition. Inhibition of CYP21A2 and CYP11B1 by MC has been reported as decreases in the consumption of PROG and DCORTICO, respectively [41]. Inhibition of CYP11B1 was estimated by the action of MC in this study, but that of CYP21A2 was not detected. In the previous experimental report, inhibitory sites by MC might have been reflected by inhibition of CYP17H activity, because CYP21A2 activity was measured as a decrease in labeled PROG.

#### 3.3.8. DZ

The mechanism of action of DZ in adrenal steroidogenesis was estimated by inhibition of CYP11A1, HSD3B2, CYP21A2, CYP11B1, and HSD17B3 (estimated inhibitions at 100 *μ*M were 58.6%, 94.1%, 96.5%, 87.2%, and 98.1%, resp.) (Figure 5(k)). DZ has been reported to inhibit HSD3B2 and CYP21A2 [46]. The results of HSD3B2 and CYP21A2 were consistent with the previous report. However, inhibitions have not been reported for CYP11A1, CYP11B1, and HSD17B3. These estimated effects of DZ on CYP11B1 and HSD17B3 were unclear, because the concentrations of ALDO and TESTO were less than the lower limit of quantification at 100 *μ*M. In addition, these enzymes act downstream of the strong action points of DZ, such as HSD3B2 and CYP21A2.

### 3.4. The Simulations and the Systematic Model Analysis

#### 3.4.1. Dynamic Sensitivity Analysis of Adrenal Steroidogenesis

**Figure 6:**
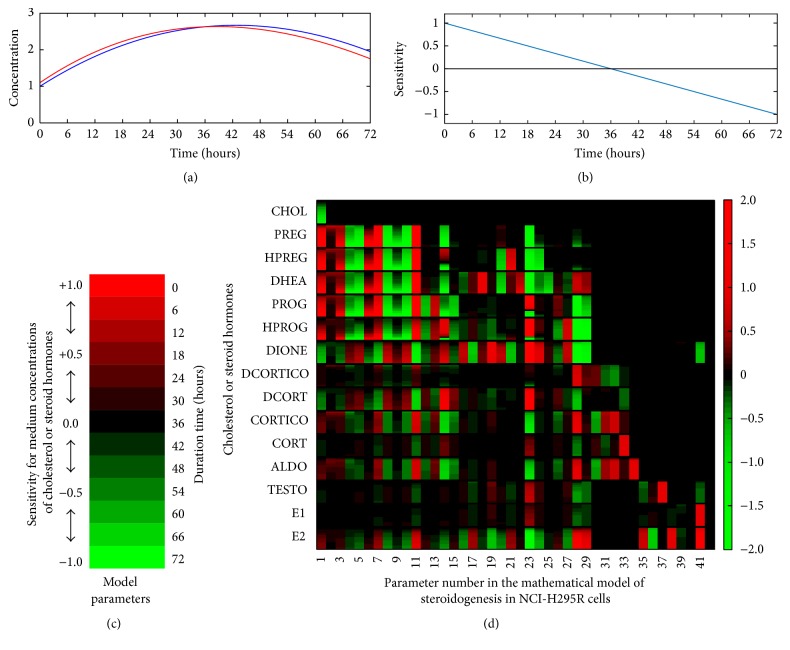
Heat-map of the global dynamic sensitivity analysis of adrenal steroid concentrations produced by NCI-H295R cells. The global dynamic sensitivity analysis is a powerful tool to comprehensively understand the dependencies of the model parameters in the mathematical model of the biological complex system. Dynamic sensitivities of model parameters in the mathematical model of adrenal steroidogenesis in NCI-H295R cells were calculated for all steroid concentrations in the culture medium every 6 h until 72 h after stimulation. To clarify the view of heat-map of global dynamic sensitivity analysis, imaginary data of the dynamics of steroid concentrations in the original model (blue line) and perturbed model for sensitivity analysis (red line) were prepared (a). Using this imaginary data, the calculated dynamic sensitivities (b) and the visualized dynamic sensitivity as one block of the heat-map (c) were shown, respectively. By the same method that explained using imaginary data, the large-scale data of the global dynamic sensitivity analysis on the mathematical model of adrenal steroidogenesis in NCI-H295R cells was comprehensively visualized as a big graph of heat-map (d). Parameter numbers in the horizontal axis are (1) *k* ^Choresterol  Transport^, (2) *k* ^CEH^, (3) *k*_*f*_^MTR^, (4) *k*_*b*_^MTR^, (5) *k*_*f*_^acc^, (6) *k*_*b*_^acc^, (7) *k*_*f*_^loc^, (8) *k*_*b*_^loc^, (9) *k*_Oxysterol  Synthesis_, (10) *K*_*m*_^CYP11A1^, (11) *V*_max_^CYP11A1^, (12) *K*_*m*A_^CYP17H^, (13) *K*_*m*B_^CYP17H^, (14) *V*_maxA_^CYP17H^, (15) *V*_maxB_^CYP17H^, (16) *V*_maxB_^CYP17H^, (17) *K*_*m*B_^CYP17L^, (18) *V*_maxA_^CYP17L^, (19) *V*_maxB_^CYP17L^, (20) *K*_*m*A_^HSD3B2^, (21) *K*_*m*B_^HSD3B2^, (22) *K*_*m*C_^HSD3B2^, (23) *V*_maxA_^HSD3B2^, (24) *V*_maxB_^HSD3B2^, (25) *V*_maxB_^HSD3B2^, (26) *K*_*m*A_^CYP21A2^, (27) *K*_*m*B_^CYP21A2^, (28) *V*_maxA_^CYP21A2^, (29) *V*_maxB_^CYP21A2^, (30) *K*_*m*A_^CYP11B1^, (31) *K*_*m*B_^CYP11B1^, (32) *V*_maxA_^CYP11B1^, (33) *V*_maxB_^CYP11B1^, (34) *k* ^CYP11B2^, (35) *K*_*m*A_^HSD17B3^, (36) *K*_*m*B_^HSD17B3^, (37) *V*_maxA_^HSD17B3^, (38) *V*_maxB_^HSD17B3^, (39) *K*_*m*A_^CYP19A1^, (40) *K*_*m*B_^CYP19A1^, (41) *V*_maxA_^CYP19A1^, and (42) *V*_maxB_^CYP19A1^. PREG: pregnenolone, HPREG: 17*α*-hydroxypregnenolone, DHEA: dehydroepiandrosterone, PROG: progesterone, HPROG: 17*α*-hydroxyprogesterone, DIONE: androstenedione, DCORTICO: 11-deoxycorticosterone, DCORT: 11-deoxycortisol, CORTICO: corticosterone, CORT: cortisol, ALDO: aldosterone, and TESTO: testosterone.

To comprehensively understand the dynamics of adrenal steroidogenesis, dynamic sensitivities were calculated for steroid concentrations secreted by NCI-H295R cells using our constructed mathematical model of steroidogenesis. The results of dynamic sensitivity analysis at 72 h of duration and 6 h of interval time are presented as a heat-map in Figure 6.

The top 10 parameters of the total area under the curve of dynamic sensitivity for cholesterol and the 14 steroids in culture medium were *V*_maxA_^HSD3B2^, *V*_max_^CYP11A1^, *V*_maxA_^CYP21A2^, *k*_*f*_^loc^, *k*_*b*_^loc^, *K*_*m*_^CYP11A1^, *V*_maxA_^CYP17H^, *k*_Cholesterol  Transport_, *k*_*f*_^acc^, and *V*_maxB_^CYP21A2^ in order from the top. Cholesterol uptake (*k*_Cholesterol  Transport_), StAR protein (*k*_*f*_^loc^), and CYP11A1 (*V*_max_^CYP11A1^), which are determining factors of the capacity for steroidogenesis, promoted the production of mineralocorticoids (DCORTICO, CORTICO, and ALDO) and restrained the synthesis of glucocorticoids (DCORT and CORT) and sex steroids (DIONE, TESTO, and E1) because of the accumulation of intermediate molecules in steroidogenesis (PREG, HPREG, DHEA, PROG, and HPROG) only by self-activation. The dynamic patterns of the intermediate molecules in steroidogenesis were mainly dependent on the activity of CYP17H and HSD3B2 with PREG as the substrate of these enzymes, in which the dynamic sensitivities of *V*_maxA_^CYP17H^ for HPREG, and HPROG and *V*_maxA_^HSD3B2^ for PROG, HPROG, and DCORTICO reversed the direction of sensitivity at 49–66 h after stimulation. The dynamic sensitivities of the maximum activities of HSD3B2 for PREG (*V*_maxA_^HSD3B2^) and CYP21A2 for PROG (*V*_maxA_^CYP21A2^) were related to all steroids at 72 h. Almost all model parameters had positive sensitivity for downstream steroids in the adrenal steroidogenic pathway and negative sensitivity for direct-binding steroids as substrates of the steroidogenic enzyme. The sensitivity of *V*_max_ in all steroidogenic enzymes was relatively higher than *K*_*m*_ for the same steroid substrate.

#### 3.4.2. Simulation of the Metabolic Balance of Adrenal Steroidogenesis Pathway

**Figure 7:**
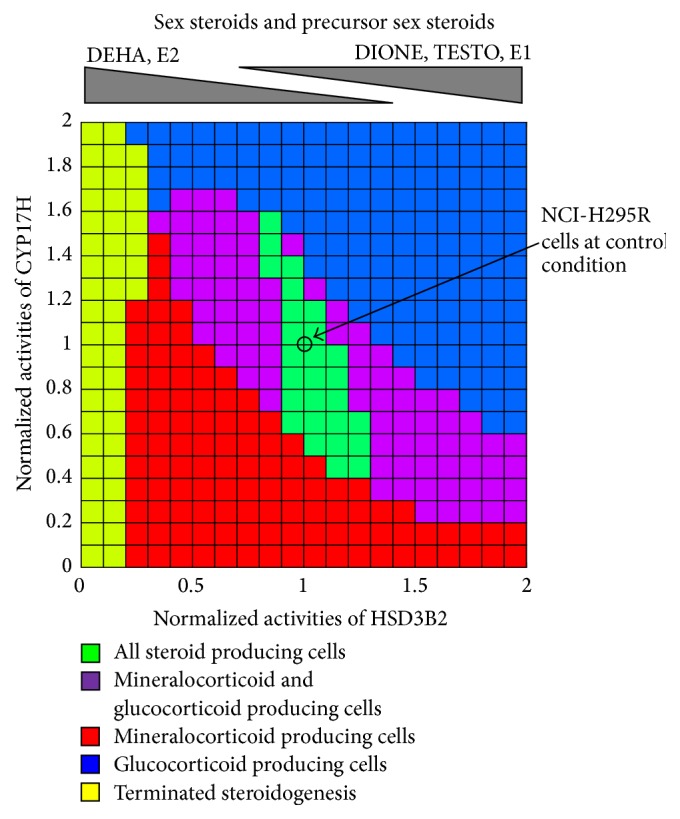
Metabolic categories of the steroidogenic cells determined by the balance of HSD3B2 and CYP17H activities. The two-dimensional parameter scanning analysis by the perturbation of two focused parameters clarifies the interaction and the relationship between the two parameters in the complex system. Functional cellular categories of steroidogenic cells were defined by the levels of mineralocorticoid (ALDO), glucocorticoids (DCORT and CORT), and androgens (DHEA and DIONE) at 72 h after stimulation. Enzymatic activities of HSD3B2 and CYP17 were normalized by standard values of the simulation model in NCI-H295R cells. Scanning ranges of HSD3B2 and CYP17 activities were 0–200%, each 10%. Green regions are all 14 steroids produced by NCI-H295R cells. Red, blue, and purple regions are mineralocorticoid producing cells, glucocorticoids-producing cells, and both corticoid-producing cells, respectively. Yellow regions are steroidogenesis of NCI-H295R cells terminated upstream of the adrenal steroidogenesis pathway.

To clearly show the property of the metabolic shift between mineralocorticoid and glucocorticoid biosynthesis, we performed two-dimensional parameter scanning of the enzymatic activities of CYP17H and HSD3B2 (Figure 7). NCI-H295R cells lost the ability to produce all steroid hormones when enzymatic activities of CYP17H and HSD3B2 were changed by over 60% and 30%, respectively. Activation of CYP17H and/or HSD3B2 induced the metabolic shift that enhanced the glucocorticoid biosynthesis and deviated from the mineralocorticoid biosynthesis. On the other hand, inhibition of CYP17H and/or HSD3B2 induced the metabolic shift that enhanced the mineralocorticoid biosynthesis and deviated from the glucocorticoid biosynthesis. Moreover, the enzymatic activity of HSD3B2 regulated the metabolic balance of sex steroids and the precursors on adrenal steroidogenesis of NCI-H295R cells. E1, TESTO, and DIONE were produced by NCI-H295R cells when activating the enzymatic activity of HSD3B2. Conversely, E2 and DHEA were produced by NCI-H295R cells when suppressing the enzymatic activity of HSD3B2. The biosynthesis of downstream steroids in adrenal steroidogenesis pathway, such as mineralocorticoids and glucocorticoids, was almost completely terminated when the enzymatic activity of HSD3B2 was decreased by over 80%.

## 4. Discussion

### 4.1. Importance of 3*β*-HSD Activity in Adrenal Steroidogenesis

Our systematic analysis using the mathematical model of adrenal steroidogenesis in NCI-H295R cells revealed that the enzymatic activity of 3*β*-HSD controls the dynamics of adrenal steroidogenesis. The activity of the StAR protein controls the net capacity of steroidogenesis in steroidogenic cells, which is the transport of cholesterol from the outer to inner mitochondrial membranes. Both the expression levels of StAR protein and mRNA are rapidly elevated in response to stimulation by tropic hormones such as ACTH [62, 63]. Another important factor in adrenal steroidogenesisis is the cholesterol side chain cleavage enzyme CYP11A1, the first rate-limiting and hormonally regulated step in the synthesis of all steroid hormones, which is conversion of cholesterol to pregnenolone in mitochondria [64]. According to our results of global sensitivity analysis (Supplementary Table  1 and Figure 6(d)), in addition to CYP11A1 and StAR proteins, 3*β*-HSD was one of the key regulators in adrenal steroidogenesis of NCI-H295R cells. And also, this result suggests that a significant regulatory mechanism in steroidogenesis pathway is very reasonable. StAR, CYP11A1, and 3*β*-HSD (isoforms 1 or 2 in humans) proteins generally respond to the same hormones that stimulate steroid production through common pathways such as cAMP signaling in adrenal glands and testes [65, 66]. Moreover, our data also support recent experimental evidence from clinical and* in vivo* studies, suggesting that the enzymatic activity of 3*β*-HSD plays an important role in the regulation of mineralocorticoid synthesis in adrenal steroidogenesis and contributes to hypertension caused by abnormal overproduction of aldosterone [67–70]. Circadian clock-deficient Cry-null mice show salt-sensitive hypertension due to abnormally high synthesis of aldosterone, which is caused by constitutively high expression of HSD3B6 mRNA and protein in the adrenal cortex [67, 68]. Recent clinical observations have revealed predominant expression of HSD3B2 mRNA and protein in tumor cells of aldosterone-producing adenoma (APA), and HSD3B1 mRNA significantly correlated with CYP11B2 mRNA levels and plasma aldosterone concentrations in APA patients [69, 70]. However, the relationship is unclear and disputed in a small-scale clinical study indicating that genetic variation in HSD3B1 affects blood pressure and hypertension in APA patients [71]. The results of our simulation study suggest that 3*β*-HSD protein (human genes are HSD3B1 and HSD3B2) is one of the determination factors for the dynamic property of adrenal steroidogenesis. Our results support the clinical evidence of Doi and colleagues [69], and we believe that the HSD3B1 enzyme has a promising potential as novel drug target for endocrine hypertension.

### 4.2. Metabolic Shift of Adrenal Steroidogenesis and the Contributions of HSD3B2 and CYP17H

The metabolic properties of adrenal steroidogenesis in NCI-H295R cells were revealed by dynamic sensitivity analysis using the mathematical model (Figure 6). Mineralocorticoids, such as DCORTICO, CORTICO, and ALDO, and intermediate steroids upstream of the adrenal steroidogenesis pathway, such as PREG, HPREG, DHEA, PROG, and HPROG, were accelerated by reactions of cholesterol import (*k*_Cholesterol  Transport_), StAR protein (*k*_*f*_^MTR^ and *k*_*f*_^loc^), and CYP11A1 (*V*_max_^CYP11A1^). On the other hand, glucocorticoids, such as DCORT and CORT, and sex hormones, such as DIONE, TESTO, and E1, were suppressed by these model parameters. Therefore, enhancement of the net adrenal steroidogenesis capacity, which supplies PREG precursor to the pathway, causes a production shift from glucocorticoids to mineralocorticoids by substrate inhibitions of CYP17H, HSD3B2, and CYP21A2 caused by accumulation of initial intermediate steroids such as PREG and PROG. Sensitivities of CYP17H (*V*_maxA_^CYP17H^) and HSD3B2 (*V*_maxA_^HSD3B2^) for the products were dynamically changed and these parameters determined the metabolic balance of downstream steroids in the adrenal steroidogenesis pathway. According to these results of dynamic sensitivity analysis of StAR, CYP11A1, CYP17H, and HSD3B2, we suggest that the enhancement of CYP17H and HSD3B2 activity during ACTH stimulation was important to shift the steroidogenic output away from ALDO biosynthesis towards CORT biosynthesis, as well as adrenal androgen production. This suggestion partially supports a comparative animal study in which molecular and cellular variations in CYP17H activity dramatically affect acute cortisol production, resulting in distinct physiological and behavioral responses [72].

Results of two-dimensional parameter scanning of the enzymatic activities of CYP17H and HSD3B2 quantitatively showed the detail of the metabolic relationship between mineralocorticoid and glucocorticoid biosynthesis (Figure 7). Particularly, the results showed that the balance of these enzymatic activities was very important for the typical function of NCI-H295R cells, namely, the ability to produce all steroid hormones. NCI-H295R cells lost this function when enzymatic activities of CYP17H and HSD3B2 were changed by over 60% and 30%, respectively. In addition, they became mineralocorticoid (ALDO) secreting cells when the enzymatic activity of CYP17H or HSD3B2 was inhibited by over 50% or glucocorticoid (DCORT and CORT) secreting cells when these enzymes were activated by over 50%. In particular, this analysis also showed that HSD3B2 was a key player in the adrenal steroidogenesis of NCI-H295R cells, because HSD3B2 inhibition by over 80% almost completely inhibited the biosynthesis of downstream steroids. The ratio of CYP17A1 to HSD3B2 mRNA expression levels has been related to several endocrine diseases with a low level in APAs [73] and high level in cortisol-producing adenomas [74]. Furthermore, the expression levels or enzymatic activities of CYP17A1 and HSD3B1 have been related to androgen production in polycystic ovary syndrome [75, 76]. These clinical studies support our simulation results indicating that the balance of enzymatic activity of CYP17H and HSD3B2 determines the shift in steroidogenic output to mineralocorticoids, glucocorticoids, or androgens.

### 4.3. Methodologies of Quantitative Mechanistic Analysis for Drug Discovery

According to our results obtained using the mathematical model of steroidogenesis in NCI-H295R cells, such as sensitivity analysis, comprehensive analysis based on systems biology is available to quantitatively estimate the mechanism of action of steroidogenic disrupting compounds from differential profiling of adrenal steroid hormones, because dynamic patterns of steroid hormones in adrenal steroidogenesis pathway are highly complex. Our proposed method of quantitative mechanistic analysis of steroidogenic inhibitors was able to predict known action sites in the adrenal steroidogenesis pathway at only one time point (72 h after drug exposure). Moreover, according to the results of sensitivity analysis (Figure 6), *V*_max_ of all steroidogenic enzymes was more sensitive than the *K*_*m*_, because the intracellular concentrations of steroid hormones were almost maintained at sufficiently high levels compared with *K*_*m*_ values of steroidogenic enzymes. These results suggested that estimation of the mechanism of action of drugs is more effective and detectable when using the influences of *V*_max_ as the searching parameters such as our proposed method. Our data showed that the proposed method based on a systems biology model is a very powerful tool for exploratory screening of steroidogenic disrupting compounds.

RCGA as a solver of parameter estimation problems in systems biology has been applied to biological network identification of gene regulatory networks and metabolic pathways and optimization of biological processes using experimentally observed time-course data [77–82]. In this study, RCGA was useful to estimate the mechanism of action of novel pharmaceutical drug candidates for adrenal steroidogenesis as a new application of RCGA in systems biology. We had two issues when applying RCGA to the quantitative mechanistic analysis of drug actions. These issues were the vast calculation cost and multimodality of quasi-optimum solutions in solving the optimization problem, because the mathematical model in systems biology consists of many equations and parameters. A proposed optimization strategy using RCGA based on REX/JGG was a highly stable and efficient calculation method for a better quasi-optimum solution than the unimodal normal distribution crossover (UNDX)/minimum generation gap (MGG) method that is well applied in the engineering field. In addition, we expanded the RCGA optimization program based on REX/JGG to a hybrid method of GA and then applied a local search as recommended by Harada and Kobayashi [28, 83]. A final optimal solution was obtained with a good convergence property. Because these problems are general in systems biology studies, we suggest that the proposed hybrid method based on REX/JGG is a very useful tool for quantitative mechanistic analysis of novel pharmaceutical drugs, not limited to steroidogenic disrupting compounds.

## 5. Conclusions

The novel mathematical model of adrenal steroidogenesis was constructed in this study, including cholesterol transport and distribution, the C_21_-steroid hormone pathway, steroid transport, and cell proliferation, which could reproduce adrenal steroidogenesis in NCI-H295R cells. According to the results of dynamic sensitivity analysis using the new model, HSD3B2 plays the most important role in the metabolic balance of adrenal steroidogenesis in NCI-H295R cells. Moreover, to quantitatively estimate mechanisms of action of adrenal toxic compounds, we analyzed differential metabolic profiles of 12 steroid hormones at 3 days after exposure to 11 adrenal toxic compounds, by using the new mathematical model and a hybrid optimization method of the RCGA and a local search (nonlinear least squares). We could estimate which steroidogenic enzymes were affected by these compounds using the hybrid optimization method. Vasculotoxic agents were estimated to have no effect according to the results obtained by our method. In terms of adrenal steroidogenic inhibitors, the predicted action sites were approximately matched to the target enzymes as reported in the literature. Thus, our computer-aided method based on a systems biology approach may be useful to analyze the mechanism of action of endocrine-disrupting compounds and to assess the human adrenal toxicity of novel pharmaceutical drugs based on steroid hormone profiling.

## Supplementary Material

Detail descriptions of a mathematical model of adrenal steroidogenesis in NCI-H295R cells were written in this supplementary document. The supplementary document includes all ordinary differentially equations (ODEs), kinetic equations of enzymatic reactions and other equations for constructing of the adrenal steroidogenesis model in this article. This adrenal steroidogenesis model consists of cell proliferation, cholesterol transport and intracellular distribution, C21-steroid hormone biosynthesis pathway, diffusional transport of steroid hormones. And also, all model parameters and all initial values of steroid hormones were described in this supplementary document that this adrenal steroidogenesis model has reproduced the experimental data of dynamic patterns of secreted steroids from NCI-H295R cells.

## Figures and Tables

**Table 1:** Adrenal toxicities and actions of tested compounds.

Test chemical	Test concentrations (*μ*M)	Pathological features of adrenal toxicity	Reported enzyme inhibitions	References
Acrylonitrile (AN)	0.1, 1, 10, and 100	Hemorrhagic adrenal necrosis	Not reported	[25]
Salinomycin (SM)	0.00001, 0.01, 0.1, 1, and 10	Damage to adrenal medullae	Not reported	[26]
Thioguanine (TG)	0.01, 1, 10, and 100	Hemorrhagic adrenal necrosis	Not reported	[25]
Fumaronitrile (FN)	0.1, 1, 10, and 100	Hemorrhagic adrenal necrosis	Not reported	[25]
Aminoglutethimide (AGT)	0.1, 1, 10, and 100	Hypertrophy, vascular degeneration	CYP11A1, CYP21A2, CYP11B1, and CYP11B2	[6, 27–30]
o,p′-DDD (DDD)	0.1, 1, 10, 25, and 100	Atrophy	CYP11A1, HSD3B2, CYP21A2, CYP11B1, and CYP11B2	[29, 31, 32]
Spironolactone (SP)	1, 10, 50, and 100	Hypertrophy	CYP17H, CYP17L, CYP11B1, and CYP11B2	[6, 33–35]
Metyrapone (MP)	0.1, 1, 10, and 100	Hypertrophy, vascular degeneration	CYP11A1, CYP11B1, and CYP11B2	[6, 29, 36–39]
Ketoconazole (KC)	0.1, 1, 10, and 100	Hypertrophy	CYP11A1, CYP17H, CYP17L, HSD3B2, CYP21A2, and CYP11B1	[6, 29, 40–43]
Miconazole (MC)	0.1, 1, 10, 25, 50, and 100	Hypertrophy	CYP11A1, CYP17H, CYP17L, CYP21A2, and CYP11B1	[41, 44, 45]
Daidzein (DZ)	0.1, 1, 10, and 100	Unknown	HSD3B2 and CYP21A2	[46]
