# Supplementary material for: Estimation of the Mechanism of Adrenal Action of Endocrine-Disrupting Compounds Using a Computational Model of Adrenal Steroidogenesis in NCI-H295R Cells
Source: J Toxicol. 2016 Feb 17;2016:4041827. doi: 10.1155/2016/4041827 (PMC4773560; doi:10.1155/2016/4041827)
Supplement: Supplementary file 1 — Detail descriptions of a mathematical model of adrenal steroidogenesis in NCI-H295R cells were written in this supplementary document. The supplementary document includes all ordinary differentially equations (ODEs), kinetic equations of enzymatic reactions and other equations for constructing of the adrenal steroidogenesis model in this article. This adrenal steroidogenesis model consists of cell proliferation, cholesterol transport and intracellular distribution, C21-steroid hormone biosynthesis pathway, diffusional transport of steroid hormones. And also, all model parameters and all initial values of steroid hormones were described in this supplementary document that this adrenal steroidogenesis model has reproduced the experimental data of dynamic patterns of secreted steroids from NCI-H295R cells. [file 4041827.f1.pdf]

A supplementary document of

Estimation of the Mechanism of Adrenal Action of Endocrine-disrupting Compounds Using a Computational Model of Adrenal Steroidogenesis in NCI-H295R Cells.

R. Saito, N. Terasaki, M. Yamazaki, N. Masutomi, N. Tsutsui, and M. Okamoto.

Journal of Toxicology, 2016: Article ID 4041827.

Contents

1. **Abbreviations**

2. **A computational model of adrenal steroidogenesis in NCI-H295R cells**
   - **Cell proliferation**
   - **Cholesterol transport and intracellular distribution**
   - **C_{21}-steroid hormone biosynthesis pathway**
   - **Diffusional transport of steroid hormones**
   - **Kinetic equations**

3. **Model parameters**
   - **Table S1. Fixed parameters and the sensitivity for fitting score**
   - **Table S2. Initial values of variable parameters**

4. **References**

1. Abbreviations

Steroid hormones

PREG: pregnenolone
HPREG: 17α-hydroxypregnenolone
DHEA: dehydroepiandrosterone
PROG: progesterone
HPROG: 17α-hydroxyprogesterone
DIONE: androstenedione
DCORTICO: 11-deoxycorticosterone
DCORT: 11-deoxycortisol
CORTICO: corticosterone
CORT: cortisol
ALDO: aldosterone
TESTO: testosterone
E1: estrone
E2: 17β-estradiol

Proteins of adrenal steroidogenic pathway

CYP11A1: cholesterol side chain cleavage enzyme
CYP17H: 17α-hydroxylase of CYP17
CYP17L: C_{17-20} lyase of CYP17
HSD3B2: 3β-hydroxysteroid dehydrogenase
CYP21A2: 21-hydroxylase
CYP11B1: 11β-hydroxylase
CYP11B2: 18-hydroxylase
HSD17B3: 17β-hydroxysteroid dehydrogenase.
CYP19A1: aromatase

2. A computational model of adrenal steroidogenesis in NCI-H295R cells

Cell proliferation

Equations to estimate the proliferation of NCI-H295R cells have been proposed by previous studies [1, 2]. In this model, the dynamics of the total number of cells ($\mathrm{N_{cell}}$) and total cell volume ($\mathrm{V_{cell}}$) were implemented by the following equations:

$$\mathrm{N_{cell}}(t) = \mathrm{N_{cell}}(-72) \cdot \exp\left[k_p \cdot (t + t_{incubation})\right] \quad \text{... (Eq. 1)}$$

$$\mathrm{V_{cell}}(t) = \mathrm{V_{indiv}} \cdot \mathrm{N_{cell}} \quad \text{... (Eq. 2)}$$

where, $\mathrm{N_{cell}}(-72)$ is the initial number of NCI-H295R cells per well before incubation (6×10^5 cells), k_p is the cell proliferation rate of NCI-H295R cells (0.00878 1/h), $t_{incubation}$ is the incubation time until stimulation (72 h), t is the culture time after stimulation, and $\mathrm{V_{indiv}}$ is the mean cell volume of individual NCI-H295R cells (1,499 μm^3).

Cholesterol transport and intracellular distribution

Cholesterol transport and the intracellular localization pathway were modified as a part of the ACTH-stimulated cortisol secretion model described by Dempsher and colleagues [3]. Intracellular cholesterol was separated into five states based on localization in the adrenal cells. There are stored cholesterol esters in the lipid droplets (CHOS), free cytosolic cholesterol (CHOC), free mitochondrial cholesterol (CHOM), free mitochondrial cholesterol close to CYP11A1 enzymes (CHON), and free mitochondrial cholesterol remote from CYP11A1 enzymes (CHOR). Cholesterols (CHOL) almost always exist as a cholesterol ester (CE) in extracellular culture medium. Therefore, imported CHOL from the culture medium is first stored in lipid droplets (transition to CHOS from medium CHOL). CHOS is transformed to free CHOL by cholesterol ester hydrolase (CEH) and distributed to the cytosolic space (transition from CHOS to CHOC). CHOC is transported into mitochondria from the cytosol by StAR protein (transition from CHOC to CHOM). CHOM is continuously translocated in the vicinity of CYP11A1 enzymes by StAR (transition from CHOM to CHON), so that CHON is available for the adrenal steroidogenesis pathway. On the other hand, CHOM also passively recedes from CYP11A1 enzymes (transition from CHOM to CHOR). Moreover, the oxysterol biosynthesis pathway was incorporated as a bypass of the steroidogenesis pathway, which was proposed by Breen et al [2]. In fact, the total mass balance of cholesterol and all steroids is not preserved in

this *in vitro* system. However, the oxysterol biosynthesis pathway and/or bypass pathway are expected to exist in NCI-H295R cells. In this model, the oxysterol biosynthesis pathway was defined to branch from CHOC to oxysterol (OXY). These variable parameters belonging to cholesterol transport and the intracellular transport system were described by the following ordinary differential equations (ODEs):

$$\frac{\mathrm{d[CHOL]_{med}}}{\mathrm{dt}} = -\frac{\mathrm{V_{cell}}}{\mathrm{V_{med}}} \cdot v_{CholesterolTransport} \quad \text{... (ODE 1)}$$

$$\frac{\mathrm{dCHOS}}{\mathrm{dt}} = \mathrm{V_{cell}} \cdot \left(v_{CholesterolTransport} - v_{CEH}\right) \quad \text{... (ODE 2)}$$

$$\frac{\mathrm{dCHOC}}{\mathrm{dt}} = \mathrm{V_{cell}} \cdot \left(v_{CEH} - v_{MTR} - v_{OxysterolSynthesis}\right) \quad \text{... (ODE 3)}$$

$$\frac{\mathrm{dCHOM}}{\mathrm{dt}} = \mathrm{V_{cell}} \cdot (v_{MTR} - v_{acc} - v_{loc}) \quad \text{... (ODE 4)}$$

$$\frac{\mathrm{dCHON}}{\mathrm{dt}} = \mathrm{V_{cell}} \cdot (v_{loc} - v_{\mathrm{CYP11A1}}) \quad \text{... (ODE 5)}$$

$$\frac{\mathrm{dCHOR}}{\mathrm{dt}} = \mathrm{V_{cell}} \cdot v_{acc} \quad \text{... (ODE 6)}$$

$$\frac{\mathrm{dOXY}}{\mathrm{dt}} = \mathrm{V_{cell}} \cdot v_{OxysterolSynthesis} \quad \text{... (ODE 7)}$$

where, $\mathrm{V_{med}}$ is the volume of culture medium (2 mL), $v_{CholesterolTransport}$ is the cholesterol import rate into the cytosolic space from the extracellular culture medium, v_{CEH} is the enzymatic reaction rate of CEH, v_{MTR} is the mitochondrial transport rate by the StAR protein, $v_{OxysterolSynthesis}$ is the reaction rate of oxysterol biosynthesis, v_{acc} is the passive diffusion rate from CYP11A1 enzymes, v_{loc} is the translocation rate close to CYP11A1 enzymes by StAR, and v_{CYP11A1} is the enzymatic reaction rate of the first adrenal steroidogenic enzyme, CYP11A1.

Net cholesterol concentration in intracellular space was calculated by a following equation:

$$\mathrm{[CHOL]_{cell}} = \frac{\mathrm{(CHOS+CHOC+CHOM+CHON+CHOR)}}{\mathrm{V_{cell}}} \quad \text{... (Eq. 3)}$$

C_{21}-steroid hormone biosynthesis pathway

The C_{21}-steroid hormone biosynthesis pathway includes 14 steroid hormones, PREG, HPREG, DHEA, PROG, HPROG, DIONE, TESTO, DCORTICO, DCORT, CORTICO, CORT, ALDO, E1 and E2, and 17 enzymatic reactions catalyzed by nine steroidogenic enzymes, CYP11A1, CYP17H, CYP17L, HSD3B2, CYP21A2, CYP11B1, CYP11B2, HSD17B3, and CYP19A1.

These variable parameters belonging to the adrenal steroidogenesis system were described by the following ODEs:

$$\mathrm{V_{cell}} \cdot \frac{\mathrm{d[PREG]_{cell}}}{\mathrm{dt}} = \frac{\mathrm{V_{cell}}^2}{\mathrm{V_{cell}}+\mathrm{V_{med}} \cdot q_{\mathrm{PREG}}} \cdot \left(v_{\mathrm{CYP11A1}} - v_{\mathrm{A,CYP17H}} - v_{\mathrm{A,HSD3B2}}\right) \quad \text{... (ODE 8)}$$

$$\mathrm{V_{cell}} \cdot \frac{\mathrm{d[HPREG]_{cell}}}{\mathrm{dt}} = \frac{\mathrm{V_{cell}}^2}{\mathrm{V_{cell}}+\mathrm{V_{med}} \cdot q_{\mathrm{HPREG}}} \cdot \left(v_{\mathrm{A,CYP17H}} - v_{\mathrm{A,CYP17L}} - v_{\mathrm{B,HSD3B2}}\right) \quad \text{... (ODE 9)}$$

$$\mathrm{V_{cell}} \cdot \frac{\mathrm{d[DHEA]_{cell}}}{\mathrm{dt}} = \frac{\mathrm{V_{cell}}^2}{\mathrm{V_{cell}}+\mathrm{V_{med}} \cdot q_{\mathrm{DHEA}}} \cdot \left(v_{\mathrm{A,CYP17L}} - v_{\mathrm{C,HSD3B2}}\right) \quad \text{... (ODE 10)}$$

$$\mathrm{V_{cell}} \cdot \frac{\mathrm{d[PROG]_{cell}}}{\mathrm{dt}} = \frac{\mathrm{V_{cell}}^2}{\mathrm{V_{cell}}+\mathrm{V_{med}} \cdot q_{\mathrm{PROG}}} \cdot \left(v_{\mathrm{A,HSD3B2}} - v_{\mathrm{B,CYP17H}} - v_{\mathrm{A,CYP21A2}}\right) \quad \text{... (ODE 11)}$$

$$\mathrm{V_{cell}} \cdot \frac{\mathrm{d[HPROG]_{cell}}}{\mathrm{dt}} = \frac{\mathrm{V_{cell}}^2}{\mathrm{V_{cell}}+\mathrm{V_{med}} \cdot q_{\mathrm{HPROG}}} \cdot \left(v_{\mathrm{B,CYP17H}} + v_{\mathrm{B,HSD3B2}} - v_{\mathrm{B,CYP17L}} - v_{\mathrm{B,CYP21A2}}\right)$$

... (ODE 12)

$$\mathrm{V_{cell}} \cdot \frac{\mathrm{d[DIONE]_{cell}}}{\mathrm{dt}} = \frac{\mathrm{V_{cell}}^2}{\mathrm{V_{cell}}+\mathrm{V_{med}} \cdot q_{\mathrm{DIONE}}} \cdot \left(v_{\mathrm{B,CYP17L}} + v_{\mathrm{C,HSD3B2}} - v_{\mathrm{A,CYP19A1}} - v_{\mathrm{A,HSD17B3}}\right)$$

... (ODE 13)

$$\mathrm{V_{cell}} \cdot \frac{\mathrm{d[TESTO]_{cell}}}{\mathrm{dt}} = \frac{\mathrm{V_{cell}}^2}{\mathrm{V_{cell}}+\mathrm{V_{med}} \cdot q_{\mathrm{TESTO}}} \cdot \left(v_{\mathrm{A,HSD17B3}} - v_{\mathrm{B,CYP19A1}}\right) \quad \text{... (ODE 14)}$$

$$\mathrm{V_{cell}} \cdot \frac{\mathrm{d[DCORTICO]_{cell}}}{\mathrm{dt}} = \frac{\mathrm{V_{cell}}^2}{\mathrm{V_{cell}}+\mathrm{V_{med}} \cdot q_{\mathrm{DCORTICO}}} \cdot \left(v_{\mathrm{A,CYP21A2}} - v_{\mathrm{A,CYP11B1}}\right) \quad \text{... (ODE 15)}$$

$$\mathrm{V_{cell}} \cdot \frac{\mathrm{d[DCORT]_{cell}}}{\mathrm{dt}} = \frac{\mathrm{V_{cell}}^2}{\mathrm{V_{cell}}+\mathrm{V_{med}} \cdot q_{\mathrm{DCORT}}} \cdot \left(v_{\mathrm{B,CYP21A2}} - v_{\mathrm{B,CYP11B1}}\right) \quad \text{... (ODE 16)}$$

$$\mathrm{V_{cell}} \cdot \frac{\mathrm{d[CORTICO]_{cell}}}{\mathrm{dt}} = \frac{\mathrm{V_{cell}}^2}{\mathrm{V_{cell}}+\mathrm{V_{med}} \cdot q_{\mathrm{CORTICO}}} \cdot \left(v_{\mathrm{A,CYP11B1}} - v_{\mathrm{CYP11B2}}\right) \quad \text{... (ODE 17)}$$

$$\mathrm{V_{cell}} \cdot \frac{\mathrm{d[CORT]_{cell}}}{\mathrm{dt}} = \frac{\mathrm{V_{cell}}^2}{\mathrm{V_{cell}}+\mathrm{V_{med}} \cdot q_{\mathrm{CORT}}} \cdot v_{\mathrm{B,CYP11B1}} \quad \text{... (ODE 18)}$$

$$\mathrm{V_{cell}} \cdot \frac{\mathrm{d[ALDO]_{cell}}}{\mathrm{dt}} = \frac{\mathrm{V_{cell}}^2}{\mathrm{V_{cell}}+\mathrm{V_{med}} \cdot q_{\mathrm{ALDO}}} \cdot v_{\mathrm{CYP11B2}} \quad \text{... (ODE 19)}$$

$$\mathrm{V_{cell}} \cdot \frac{\mathrm{d[E1]_{cell}}}{\mathrm{dt}} = \frac{\mathrm{V_{cell}}^2}{\mathrm{V_{cell}}+\mathrm{V_{med}} \cdot q_{\mathrm{E1}}} \cdot \left(v_{\mathrm{A,CYP19A1}} - v_{\mathrm{B,HSD17B3}}\right) \quad \text{... (ODE 20)}$$

$$\mathrm{V_{cell}} \cdot \frac{\mathrm{d[E2]_{cell}}}{\mathrm{dt}} = \frac{\mathrm{V_{cell}}^2}{\mathrm{V_{cell}}+\mathrm{V_{med}} \cdot q_{\mathrm{E2}}} \cdot \left(v_{\mathrm{B,HSD17B3}} + v_{\mathrm{B,CYP19A1}}\right) \quad \text{... (ODE 21)}$$

where, q_{x} (dimensionless) is the equilibrium constant of steroid hormone x between the cytosol and extracellular culture medium and v_{X} (μM/h) is the enzymatic reaction rate of

steroidogenic enzyme X.

Diffusional transport of steroid hormones

Descriptions of all passive transport reactions of steroid hormones were based on the quasi-equilibrium reaction. Therefore, steroid concentrations in the culture medium were calculated from each cytosolic concentration and equilibrium constant.

$$[\mathrm{PREG}]_{\mathrm{med}} = q_{\mathrm{PREG}} \cdot [\mathrm{PREG}]_{\mathrm{cell}} \quad \text{... (Eq. 4)}$$

$$[\mathrm{HPREG}]_{\mathrm{med}} = q_{\mathrm{HPREG}} \cdot [\mathrm{HPREG}]_{\mathrm{cell}} \quad \text{... (Eq. 5)}$$

$$[\mathrm{DHEA}]_{\mathrm{med}} = q_{\mathrm{DHEA}} \cdot [\mathrm{DHEA}]_{\mathrm{cell}} \quad \text{... (Eq. 6)}$$

$$[\mathrm{PROG}]_{\mathrm{med}} = q_{\mathrm{PROG}} \cdot [\mathrm{PROG}]_{\mathrm{cell}} \quad \text{... (Eq. 7)}$$

$$[\mathrm{HPROG}]_{\mathrm{med}} = q_{\mathrm{HPROG}} \cdot [\mathrm{HPROG}]_{\mathrm{cell}} \quad \text{... (Eq. 8)}$$

$$[\mathrm{DIONE}]_{\mathrm{med}} = q_{\mathrm{DIONE}} \cdot [\mathrm{DIONE}]_{\mathrm{cell}} \quad \text{... (Eq. 9)}$$

$$[\mathrm{TESTO}]_{\mathrm{med}} = q_{\mathrm{TESTO}} \cdot [\mathrm{TESTO}]_{\mathrm{cell}} \quad \text{... (Eq. 10)}$$

$$[\mathrm{DCORTICO}]_{\mathrm{med}} = q_{\mathrm{DCORTICO}} \cdot [\mathrm{DCORTICO}]_{\mathrm{cell}} \quad \text{... (Eq. 11)}$$

$$[\mathrm{DCORT}]_{\mathrm{med}} = q_{\mathrm{DCORT}} \cdot [\mathrm{DCORT}]_{\mathrm{cell}} \quad \text{... (Eq. 12)}$$

$$[\mathrm{CORTICO}]_{\mathrm{med}} = q_{\mathrm{CORTICO}} \cdot [\mathrm{CORTICO}]_{\mathrm{cell}} \quad \text{... (Eq. 13)}$$

$$[\mathrm{CORT}]_{\mathrm{med}} = q_{\mathrm{CORT}} \cdot [\mathrm{CORT}]_{\mathrm{cell}} \quad \text{... (Eq. 14)}$$

$$[\mathrm{ALDO}]_{\mathrm{med}} = q_{\mathrm{ALDO}} \cdot [\mathrm{ALDO}]_{\mathrm{cell}} \quad \text{... (Eq. 15)}$$

$$[\mathrm{E1}]_{\mathrm{med}} = q_{\mathrm{E1}} \cdot [\mathrm{E1}]_{\mathrm{cell}} \quad \text{... (Eq. 16)}$$

$$[\mathrm{E2}]_{\mathrm{med}} = q_{\mathrm{E2}} \cdot [\mathrm{E2}]_{\mathrm{cell}} \quad \text{... (Eq. 17)}$$

Kinetic equations

In this mathematical model of adrenal steroidogenesis in NCI-H295R cells, the flux velocities of molecular transportation and enzymatic reaction rates of steroidogenic enzymes were defined based on the first-order reaction and rapid-equilibrium enzyme kinetics, respectively, by the following equations:

$$v_{CholesterolTransport} = k^{CholesterolTransport} \cdot [\mathrm{CHOL}]_{\mathrm{med}} \cdot \frac{\mathrm{V_{med}}}{\mathrm{V_{cell}}} \quad \text{... (Eq. 18)}$$

$$v_{CEH} = k^{CEH} \cdot [\mathrm{CHOS}]_{\mathrm{cell}} \quad \text{... (Eq. 19)}$$

$$v_{OxysterolSynthesis} = k^{OxysterolSynthesis} \cdot [\mathrm{CHOC}]_{\mathrm{cell}} \quad \text{... (Eq. 20)}$$

$$v_{MTR} = k_f^{MTR} \cdot [\mathrm{CHOC}]_{\mathrm{cell}} - k_b^{MTR} \cdot [\mathrm{CHOM}]_{\mathrm{cell}} \quad \text{... (Eq. 21)}$$

$$v_{acc} = k_f^{acc} \cdot [\mathrm{CHOM}]_{\mathrm{cell}} - k_b^{acc} \cdot [\mathrm{CHOR}]_{\mathrm{cell}} \quad \text{... (Eq. 22)}$$

$$v_{loc} = k_f^{loc} \cdot [\mathrm{CHOM}]_{\mathrm{cell}} - k_b^{loc} \cdot [\mathrm{CHON}]_{\mathrm{cell}} \quad \text{... (Eq. 23)}$$

$$v_{\mathrm{CYP11A1}} = \frac{V_{\mathrm{max}}^{\mathrm{CYP11A1}} \cdot [\mathrm{CHON}]_{\mathrm{cell}}}{[\mathrm{CHON}]_{\mathrm{cell}} + K_m^{\mathrm{CYP11A1}}} \quad \text{... (Eq. 24)}$$

$$v_{\mathrm{A,CYP17H}} = \frac{V_{\mathrm{maxA}}^{\mathrm{CYP17H}} \frac{[\mathrm{PREG}]_{\mathrm{cell}}}{K_{m\mathrm{A}}^{\mathrm{CYP17H}}}}{1 + \frac{[\mathrm{PREG}]_{\mathrm{cell}}}{K_{m\mathrm{A}}^{\mathrm{CYP17H}}} + \frac{[\mathrm{PROG}]_{\mathrm{cell}}}{K_{m\mathrm{B}}^{\mathrm{CYP17H}}}} \quad \text{... (Eq. 25)}$$

$$v_{\mathrm{B,CYP17H}} = \frac{V_{\mathrm{maxB}}^{\mathrm{CYP17H}} \cdot \frac{[\mathrm{PROG}]_{\mathrm{cell}}}{K_{m\mathrm{B}}^{\mathrm{CYP17H}}}}{1 + \frac{[\mathrm{PREG}]_{\mathrm{cell}}}{K_{m\mathrm{A}}^{\mathrm{CYP17H}}} + \frac{[\mathrm{PROG}]_{\mathrm{cell}}}{K_{m\mathrm{B}}^{\mathrm{CYP17H}}}} \quad \text{... (Eq. 26)}$$

$$v_{\mathrm{A,CYP17L}} = \frac{V_{\mathrm{maxA}}^{\mathrm{CYP17L}} \cdot \frac{[\mathrm{HPREG}]_{\mathrm{cell}}}{K_{m\mathrm{A}}^{\mathrm{CYP17L}}}}{1 + \frac{[\mathrm{HPREG}]_{\mathrm{cell}}}{K_{m\mathrm{A}}^{\mathrm{CYP17L}}} + \frac{[\mathrm{HPROG}]_{\mathrm{cell}}}{K_{m\mathrm{B}}^{\mathrm{CYP17L}}}} \quad \text{... (Eq. 27)}$$

$$v_{\mathrm{B,CYP17L}} = \frac{V_{\mathrm{maxB}}^{\mathrm{CYP17L}} \cdot \frac{[\mathrm{HPROG}]_{\mathrm{cell}}}{K_{m\mathrm{B}}^{\mathrm{CYP17L}}}}{1 + \frac{[\mathrm{HPREG}]_{\mathrm{cell}}}{K_{m\mathrm{A}}^{\mathrm{CYP17L}}} + \frac{[\mathrm{HPROG}]_{\mathrm{cell}}}{K_{m\mathrm{B}}^{\mathrm{CYP17L}}}} \quad \text{... (Eq. 28)}$$

$$v_{\mathrm{A,HSD3B2}} = \frac{V_{\mathrm{maxA}}^{\mathrm{HSD3B2}} \cdot \frac{[\mathrm{PREG}]_{\mathrm{cell}}}{K_{m\mathrm{A}}^{\mathrm{HSD3B2}}}}{1 + \frac{[\mathrm{PREG}]_{\mathrm{cell}}}{K_{m\mathrm{A}}^{\mathrm{HSD3B2}}} + \frac{[\mathrm{HPREG}]_{\mathrm{cell}}}{K_{m\mathrm{B}}^{\mathrm{HSD3B2}}} + \frac{[\mathrm{DHEA}]_{\mathrm{cell}}}{K_{m\mathrm{C}}^{\mathrm{HSD3B2}}}} \quad \text{... (Eq. 29)}$$

$$v_{\mathrm{B,HSD3B2}} = \frac{V_{\mathrm{maxB}}^{\mathrm{HSD3B2}} \cdot \frac{[\mathrm{HPREG}]_{\mathrm{cell}}}{K_{m\mathrm{B}}^{\mathrm{HSD3B2}}}}{1 + \frac{[\mathrm{PREG}]_{\mathrm{cell}}}{K_{m\mathrm{A}}^{\mathrm{HSD3B2}}} + \frac{[\mathrm{HPREG}]_{\mathrm{cell}}}{K_{m\mathrm{B}}^{\mathrm{HSD3B2}}} + \frac{[\mathrm{DHEA}]_{\mathrm{cell}}}{K_{m\mathrm{C}}^{\mathrm{HSD3B2}}}} \quad \text{... (Eq. 30)}$$

$$v_{\mathrm{C,HSD3B2}} = \frac{V_{\mathrm{maxC}}^{\mathrm{HSD3B2}} \cdot \frac{[\mathrm{DHEA}]_{\mathrm{cell}}}{K_{m\mathrm{C}}^{\mathrm{HSD3B2}}}}{1 + \frac{[\mathrm{PREG}]_{\mathrm{cell}}}{K_{m\mathrm{A}}^{\mathrm{HSD3B2}}} + \frac{[\mathrm{HPREG}]_{\mathrm{cell}}}{K_{m\mathrm{B}}^{\mathrm{HSD3B2}}} + \frac{[\mathrm{DHEA}]_{\mathrm{cell}}}{K_{m\mathrm{C}}^{\mathrm{HSD3B2}}}} \quad \text{... (Eq. 31)}$$

$$v_{\mathrm{A,CYP21A1}} = \frac{V_{\mathrm{maxA}}^{\mathrm{CYP21A1}} \cdot \frac{[\mathrm{PROG}]_{\mathrm{cell}}}{K_{m\mathrm{A}}^{\mathrm{CYP21A1}}}}{1 + \frac{[\mathrm{PROG}]_{\mathrm{cell}}}{K_{m\mathrm{A}}^{\mathrm{CYP21A1}}} + \frac{[\mathrm{HPROG}]_{\mathrm{cell}}}{K_{m\mathrm{B}}^{\mathrm{CYP21A1}}}} \quad \text{... (Eq. 32)}$$

$$v_{\mathrm{B,CYP21A1}} = \frac{V_{\mathrm{maxB}}^{\mathrm{CYP21A1}} \cdot \frac{[\mathrm{HPROG}]_{\mathrm{cell}}}{K_{m\mathrm{B}}^{\mathrm{CYP21A1}}}}{1 + \frac{[\mathrm{PROG}]_{\mathrm{cell}}}{K_{m\mathrm{A}}^{\mathrm{CYP21A1}}} + \frac{[\mathrm{HPROG}]_{\mathrm{cell}}}{K_{m\mathrm{B}}^{\mathrm{CYP21A1}}}} \quad \text{... (Eq. 33)}$$

$$v_{\mathrm{A,CYP11B1}} = \frac{V_{\mathrm{maxA}}^{\mathrm{CYP11B1}} \cdot \frac{[\mathrm{DCORTICO}]_{\mathrm{cell}}}{K_{m\mathrm{A}}^{\mathrm{CYP11B1}}}}{1 + \frac{[\mathrm{DCORTICO}]_{\mathrm{cell}}}{K_{m\mathrm{A}}^{\mathrm{CYP11B1}}} + \frac{[\mathrm{DCORT}]_{\mathrm{cell}}}{K_{m\mathrm{B}}^{\mathrm{CYP11B1}}}} \quad \text{... (Eq. 34)}$$

$$v_{\mathrm{B,CYP11B1}} = \frac{V_{\mathrm{maxB}}^{\mathrm{CYP11B1}} \cdot \frac{[\mathrm{DCORT}]_{\mathrm{cell}}}{K_{m\mathrm{B}}^{\mathrm{CYP11B1}}}}{1 + \frac{[\mathrm{DCORTICO}]_{\mathrm{cell}}}{K_{m\mathrm{A}}^{\mathrm{CYP11B1}}} + \frac{[\mathrm{DCORT}]_{\mathrm{cell}}}{K_{m\mathrm{B}}^{\mathrm{CYP11B1}}}} \quad \text{... (Eq. 35)}$$

$$v_{\mathrm{CYP11B2}} = k^{\mathrm{CYP11B2}} \cdot [\mathrm{CORTICO}]_{\mathrm{cell}} \quad \text{... (Eq. 36)}$$

$$v_{\mathrm{A,HSD17B3}} = \frac{V_{\mathrm{maxA}}^{\mathrm{HSD17B3}} \cdot \frac{[\mathrm{DIONE}]_{\mathrm{cell}}}{K_{m\mathrm{A}}^{\mathrm{HSD17B3}}}}{1 + \frac{[\mathrm{DIONE}]_{\mathrm{cell}}}{K_{m\mathrm{A}}^{\mathrm{HSD17B3}}} + \frac{[\mathrm{E1}]_{\mathrm{cell}}}{K_{m\mathrm{B}}^{\mathrm{HSD17B3}}}} \quad \text{... (Eq. 37)}$$

$$v_{\mathrm{B,HSD17B3}} = \frac{V_{\mathrm{maxB}}^{\mathrm{HSD17B3}} \cdot \frac{[\mathrm{E1}]_{\mathrm{cell}}}{K_{m\mathrm{B}}^{\mathrm{HSD17B3}}}}{1 + \frac{[\mathrm{DIONE}]_{\mathrm{cell}}}{K_{m\mathrm{A}}^{\mathrm{HSD17B3}}} + \frac{[\mathrm{E1}]_{\mathrm{cell}}}{K_{m\mathrm{B}}^{\mathrm{HSD17B3}}}} \quad \text{... (Eq. 38)}$$

$$v_{\mathrm{A,CYP19A1}} = \frac{V_{\mathrm{maxA}}^{\mathrm{CYP19A1}} \cdot \frac{[\mathrm{DIONE}]_{\mathrm{cell}}}{K_{m\mathrm{A}}^{\mathrm{CYP19A1}}}}{1 + \frac{[\mathrm{DIONE}]_{\mathrm{cell}}}{K_{m\mathrm{A}}^{\mathrm{CYP19A1}}} + \frac{[\mathrm{TESTO}]_{\mathrm{cell}}}{K_{m\mathrm{B}}^{\mathrm{CYP19A1}}}} \quad \text{... (Eq. 39)}$$

$$v_{\mathrm{B,CYP19A1}} = \frac{V_{\mathrm{maxB}}^{\mathrm{CYP19A1}} \cdot \frac{[\mathrm{TESTO}]_{\mathrm{cell}}}{K_{m\mathrm{B}}^{\mathrm{CYP19A1}}}}{1 + \frac{[\mathrm{DIONE}]_{\mathrm{cell}}}{K_{m\mathrm{A}}^{\mathrm{CYP19A1}}} + \frac{[\mathrm{TESTO}]_{\mathrm{cell}}}{K_{m\mathrm{B}}^{\mathrm{CYP19A1}}}} \quad \text{... (Eq. 40)}$$

where k^{X} (1/h) is the rate constant of the first order reaction for metabolic flux X, $V_{\mathrm{maxA}}^{\mathrm{X}}$ (μM/h) is the maximum velocity of enzyme X for substrate A, and $K_{m\mathrm{A}}^{\mathrm{X}}$ is the equilibrium dissociation constant of enzyme X for substrate A.

3. Model parameters

All static parameters in this model are described in Supplementary Table 1. Rate constants and the maximum velocity were estimated by fitting to experimental time-course data of the concentrations of cholesterol and all steroids. Equilibrium dissociation constants were extracted from previous biochemical studies. Equilibrium constants of each steroid transport were estimated in previous studies by Breen and colleagues [1, 2]. All initial values of variable parameters in this model are described in Supplementary Table 2. Initial values of cholesterol and the 14 steroid concentrations were used in each experimentally measured value, and every steroid concentration was assumed to rapidly reach the equilibrium state between the culture medium and intracellular space.

Table S1. Fixed parameters in the adrenal steroidgenesis model of NCI-H295R cells

Parameter number	Model parameter	Optimized value	Units	Sensitivity for the fitting objective function	Reference
1	$k^{CholesterolTransport}$	0.0197596	1/h	4.6234	optimized
2	k^{CEH}	0.302850	1/h	0.0331	optimized
3	k_f^{MTR}	11.7203	1/h	1.4953	optimized
4	k_b^{MTR}	202.531	1/h	1.7737	optimized
5	k_f^{acc}	5.17768	1/h	3.1793	optimized
6	k_b^{acc}	0.0404461	1/h	1.8855	optimized
7	k_f^{loc}	9.97234	1/h	8.0399	optimized
8	k_b^{loc}	2339.88	1/h	5.8004	optimized
9	$k_{OxysterolSynthesis}$	0.0654339	1/h	1.3421	optimized
10	K_m^{CYP11A1}	0.5	μM	5.8000	(4)
11	$V_{\mathrm{max}}^{\mathrm{CYP11A1}}$	675.701	μM/h	22.4750	optimized
12	$K_{m\mathrm{A}}^{\mathrm{CYP17H}}$	0.25	μM	0.2342	(5)
13	$K_{m\mathrm{B}}^{\mathrm{CYP17H}}$	0.45	μM	0.2570	(5)
14	$V_{\mathrm{maxA}}^{\mathrm{CYP17H}}$	108.063	μM/h	3.8388	optimized
15	$V_{\mathrm{maxB}}^{\mathrm{CYP17H}}$	1050.21	μM/h	0.2985	optimized
16	$K_{m\mathrm{A}}^{\mathrm{CYP17L}}$	0.270	μM	0.1274	(6)
17	$K_{m\mathrm{B}}^{\mathrm{CYP17L}}$	0.525	μM	0.1597	(7)

18	$V_{\mathrm{maxA}}^{\mathrm{CYP17L}}$	10.8710	μM/h	0.2107	optimized
19	$V_{\mathrm{maxB}}^{\mathrm{CYP17L}}$	69.7546	μM/h	0.3717	optimized
20	$K_{m\mathrm{A}}^{\mathrm{HSD3B2}}$	2.8	μM	0.3626	(8)
21	$K_{m\mathrm{B}}^{\mathrm{HSD3B2}}$	3.5	μM	0.4001	(8)
22	$K_{m\mathrm{C}}^{\mathrm{HSD3B2}}$	3.7	μM	0.0929	(8)
23	$V_{\mathrm{maxA}}^{\mathrm{HSD3B2}}$	235.152	μM/h	30.9490	optimized
24	$V_{\mathrm{maxB}}^{\mathrm{HSD3B2}}$	1901.65	μM/h	0.6361	optimized
25	$V_{\mathrm{maxC}}^{\mathrm{HSD3B2}}$	525.917	μM/h	0.1046	optimized
26	$K_{m\mathrm{A}}^{\mathrm{CYP21A2}}$	1.5	μM	0.1652	(9)
27	$K_{m\mathrm{B}}^{\mathrm{CYP21A2}}$	1.6	μM	0.3853	(9)
28	$V_{\mathrm{maxA}}^{\mathrm{CYP21A2}}$	213.911	μM/h	7.0023	optimized
29	$V_{\mathrm{maxB}}^{\mathrm{CYP21A2}}$	544.496	μM/h	1.9225	optimized
30	$K_{m\mathrm{A}}^{\mathrm{CYP11B1}}$	2.50	μM	0.1095	(10)
31	$K_{m\mathrm{B}}^{\mathrm{CYP11B1}}$	0.882	μM	0.1042	(11)
32	$V_{\mathrm{maxA}}^{\mathrm{CYP11B1}}$	214.848	μM/h	0.2427	optimized
33	$V_{\mathrm{maxB}}^{\mathrm{CYP11B1}}$	36.9996	μM/h	0.1781	optimized
34	k^{CYP11B2}	0.0698923	1/h	0.1634	optimized
35	$K_{m\mathrm{A}}^{\mathrm{HSD17B3}}$	0.7	μM	0.1022	(12)
36	$K_{m\mathrm{B}}^{\mathrm{HSD17B3}}$	3.3	μM	0.1111	(12)
37	$V_{\mathrm{maxA}}^{\mathrm{HSD17B3}}$	0.81059	μM/h	0.1666	optimized
38	$V_{\mathrm{maxB}}^{\mathrm{HSD17B3}}$	6.15932	μM/h	0.1769	optimized
39	$K_{m\mathrm{A}}^{\mathrm{CYP19A1}}$	0.215	μM	0.0035	(13)
40	$K_{m\mathrm{B}}^{\mathrm{CYP19A1}}$	0.370	μM	0.0003	(13)
41	$V_{\mathrm{maxA}}^{\mathrm{CYP19A1}}$	10.0091	μM/h	0.7052	optimized
42	$V_{\mathrm{maxB}}^{\mathrm{CYP19A1}}$	1.0e-6	μM/h	0.0000	optimized
43	q_{PREG}	0.0129	dimensionless	---	(2)
44	q_{HPREG}	0.0605	dimensionless	---	(2)
45	q_{DHEA}	0.0377	dimensionless	---	(2)
46	q_{PROG}	0.0052	dimensionless	---	(2)
47	q_{HPROG}	0.0212	dimensionless	---	(2)
48	q_{DIONE}	0.0400	dimensionless	---	(2)
49	q_{DCORTICO}	0.0412	dimensionless	---	(2)
50	q_{DCORT}	0.0422	dimensionless	---	(2)
51	q_{CORTICO}	0.0558	dimensionless	---	(2)

52	q_{CORT}	0.0676	dimensionless	---	(2)
53	q_{ALDO}	0.0911	dimensionless	---	(2)
54	q_{TESTO}	0.0443	dimensionless	---	(2)
55	q_{E1}	0.0267	dimensionless	---	(2)
56	q_{E2}	0.0351	dimensionless	---	(2)

Table S2. Initial values of variable parameters in the adrenal steroidogenesis model of NCI-H295R cells.

Compartment	Molecular parameter name	Initial value	Unit
Culture medium	CHOL	81.1	µM
Culture medium	PREG	0.00085	µM
Culture medium	HPREG	0.06945	µM
Culture medium	DHEA	0.0	µM
Culture medium	PROG	0.00003	µM
Culture medium	HPROG	0.0	µM
Culture medium	DIONE	0.00080	µM
Culture medium	DCORTICO	0.0	µM
Culture medium	DCORT	0.0	µM
Culture medium	CORTICO	0.00011	µM
Culture medium	CORT	0.00003	µM
Culture medium	ALDO	0.00091	µM
Culture medium	TESTO	0.00080	µM
Culture medium	E1	0.00011	µM
Culture medium	E2	0.00121	µM
Intracellular space	OXY	0.0	nmol
Intracellular space	CHOS	16.08	nmol
Intracellular space	CHOC	0.3503	nmol
Intracellular space	CHOM	0.02943	nmol
Intracellular space	CHON	0.0006431	nmol
Intracellular space	CHOR	0.6350	nmol
Intracellular space	PREG	0.008580	nmol
Intracellular space	HPREG	0.0	nmol
Intracellular space	DHEA	0.003165	nmol

Intracellular space	PROG	0.00002892	nmol
Intracellular space	HPROG	0.00009361	nmol
Intracellular space	DIONE	0.002115	nmol
Intracellular space	DCORTICO	0.0007598	nmol
Intracellular space	DCORT	0.06871	nmol
Intracellular space	CORTICO	0.002065	nmol
Intracellular space	CORT	0.003114	nmol
Intracellular space	ALDO	0.0	nmol
Intracellular space	TESTO	0.0	nmol
Intracellular space	E1	0.001895	nmol
Intracellular space	E2	0.0003870	nmol

4. References

1. Breen MS, Breen M, Terasaki N, Yamazaki M, Conolly RB. Computational model of steroidogenesis in human H295R cells to predict biochemical response to endocrine-active chemicals: model development for metyrapone. Environ Health Perspect. 2010; 118(2):265-72.
2. Breen M, Breen MS, Terasaki N, Yamazaki M, Lloyd AL, Conolly RB. Mechanistic computational model of steroidogenesis in H295R cells: role of oxysterols and cell proliferation to improve predictability of biochemical response to endocrine active chemical-metyrapone. Toxicol Sci. 2011; 123(1):80-93.
3. Dempsher DP, Gann DS, Phair RD. A mechanistic model of ACTH-stimulated cortisol secretion. Am J Physiol. 1984; 246(4 Pt 2):R587-96.
4. Warne PA, Greenfield NJ, Lieberman S. Modulation of the kinetics of cholesterol side-chain cleavage by an activator and by an inhibitor isolated from the cytosol of the cortex of bovine adrenals. Proc Natl Acad Sci U S A. 1983; 80(7):1877-81.
5. Beaudoin C, Lavallee B, Tremblay Y, et al. Modulation of 17alpha-hydroxylase/17,20-lyase activity of guinea pig cytochrome P450c17 by site-directed mutagenesis. DNA Cell Biol. 1998; 17(8):707-15.
6. Rosa S, Steigert M, Lang-Muritano M, I'Allemand D, Schoenle EJ, Biason-Lauber A. Clinical, genetic and functional characteristics of three novel CYP17A1 mutations causing combined 17alpha-hydroxylase/17,20-lyase deficiency. Horm Res Paediatr. 2010; 73(3):198-204.

7. Dalla Valle L, Ramina A, Vianello S, Belvedere P, Colombo L. Kinetic analysis of duodenal and testicular cytochrome P450c17 in the rat. J Steroid Biochem Med Biol. 1996; 58(5-6):577-84.
8. Thomas JL, Mason JI, Brandt S, Spencer BR Jr, Norris W. Structure/function relationships responsible for the kinetic differences between human type 1 and type 2 3beta-hydroxysteroid dehydrogenase and for the catalysis of the type 1 activity. J Biol Chem. 2002; 277(45): 42795-801.
9. Riepe FG, Hiort O, Sippell WG, Krone N, Holterhus PM. Functional and structural consequences of a novel point mutation in the CYP21A2 gene causing congenital adrenal hyperplasia: potential relevance of helix C for P450 oxidoreductase-21-hydroxylase interaction. J Clin Endocrinol Metab. 2008; 93(7):2891-5.
10. Fisher A, Fraser R, Mc Connell J, Davies E. Amino acid residue 147 of human aldosterone synthase and 11beta-hydroxylase plays a key role in 11beta-hydroxylation. J Clin Endocrinol Metab. 2000; 85(3):1261-6.
11. LaSala D, Shibanaka Y, Jeng AY. Coexpression of CYP11B2 or CYP11B1 with adrenodoxin and adrenodoxin reductase for assessing the potency and selectivity of aldosterone synthase inhibitors. Anal Biochem. 2009; 394(1):56-61.
12. Baker ME. Unusual evolution of 11beta- and 17beta-hydroxysteroid and retinol dehydrogenases. BioEssays. 1996; 18(1):63-70.
13. Corbin CJ, Mapes SM, Lee YM, Conley AJ. Structural and functional differences among purified recombinant mammalian aromatases: glycosylation, N-terminal sequence and kinetic analysis of human, bovine and the porcine placental and gonadal isozymes. Mol Cell Endocrinol. 2003; 206(1-2):147-57.
